# Dual‐Network Protein Hydrogels Promote Rapid Hemostasis and Immune‐Regulated Scarless Tissue Regeneration

**DOI:** 10.1002/advs.75324

**Published:** 2026-04-20

**Authors:** Xiaomei Li, Guorui Zhang, Xinyue Wang, Zhongyu Liu, Ling Yang, Yuhan Bao, Min Wei, Yonglin Chen, Yanfei Ma, Wenbo Sheng, Bo Yu, Bin Li

**Affiliations:** ^1^ The First Clinical Medical College of Lanzhou University Lanzhou China; ^2^ State Key Laboratory of Solid Lubrication Lanzhou Institute of Chemical Physics Chinese Academy of Sciences Lanzhou China; ^3^ Shandong Laboratory of Advanced Materials and Green Manufacturing At Yantai Shandong China

**Keywords:** hydrogel, protein, scarless tissue regeneration, wound healing

## Abstract

Bioadhesive hydrogels hold great promise for rapid hemostasis and tissue repair, yet their clinical translation remains limited by inadequate mechanical robustness, transient adhesion, and uncontrolled fibrotic healing, which can cause pain, prolong wound healing, and unsightly scars. Here, we report an injectable, dual‐network fibrin–dextran hydrogel that integrates the intrinsic bioactivity of fibrin with the mechanical tunability of methacrylated dextran (Dex‐MA) and the strong adhesion to wet, slippery wound skin mediated by dopamine methacrylamide (DMA). The hydrogel undergoes dual enzymatic and redox‐triggered crosslinking—via thrombin and dithiothreitol to form an adaptive network exhibiting programmable stiffness and dynamic adhesion. Beyond its rapid hemostatic function, the hydrogel demonstrates potent anti‐inflammatory and anti‐scar formation activity, achieved through reactive oxygen species (ROS) scavenging and macrophage polarization toward a pro‐regenerative M2 phenotype. Transcriptomic analysis (RNA‐seq) further reveals activation of antioxidant defense and suppression of TGF‐β‐driven fibrotic pathways, thereby minimizing collagen hyper‐deposition and scar tissue formation. In rat liver and tail amputation models, the hydrogel significantly reduced blood loss, shortened bleeding time, and promoted scar‐free wound closure. This study establishes a bioinspired hydrogel platform that integrates hemostasis, immunomodulation, and anti‐fibrotic healing, offering a generalized and sustainable strategy for designing next‐generation bioadhesive materials for scarless tissue regeneration.

## Graphical abstract

A dual‐network protein hydrogel substantially improves hemostasis and scar‐free healing.The adaptive network maintains intimate tissue contact while providing strong wet adhesion, tunable mechanics, and controlled degradation. Concurrent ROS scavenging and M2 polarization suppress fibrotic pathways, preventing scar formation. This strategy highlights the potential of multifunctional, mechanically compliant hydrogels to create immunomodulatory, anti‐fibrotic biointerfaces for scar‐free tissue repairing.

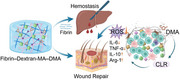

## 1 Introduction

Uncontrolled hemorrhage, particularly after traumatic injury, remains a leading cause of preventable death and disability worldwide [1, 2, 3, 4]. Rapid and effective hemostasis during the early phase of injury is therefore critical for saving lives [5, 6, 7]. The skin, as the body's primary physical and immunological barrier, is frequently subject to mechanical and thermal injuries. Although cutaneous wounds eventually close spontaneously, they rarely restore the skin's native architecture [8, 9]. Healing often culminates in fibrotic scar tissue that lacks essential appendages, such as hair follicles and sweat glands. Wound repair proceeds through four coordinated stages—hemostasis, inflammation, proliferation, and remodeling [10, 11, 12]. However, excessive reactive oxygen species (ROS) can disrupt this process, leading to prolonged inflammation, impaired collagen deposition, impaired angiogenesis, and pathological scarring. These complications impose a substantial clinical and socioeconomic burden [13, 14, 15].

Effective wound management thus requires a multifunctional strategy capable of addressing several interrelated challenges: (i) achieving rapid hemostasis to prevent life‐threatening blood loss, (ii) maintaining a moist and sterile microenvironment to facilitate repair, and (iii) modulating oxidative and inflammatory stress and regulating immune cell activity [16, 17, 18]. Traditional dressings such as gauze and gelatin sponges offer passive coverage but lack bioactivity and mechanical resilience, leading to secondary injury, infection, and delayed healing [19, 20, 21, 22]. Hence, next‐generation wound dressings that combine bioactivity, adaptability, and mechanical robustness are urgently needed, particularly for complex trauma and emergency care [23, 24].

Hydrogels are ideal candidates due to their high water content, tunable degradability, tissue conformity, and structural and functional similarity to extracellular matrix (ECM) [9, 25, 26, 27, 28]. Their hydrated networks maintain a moist environment that accelerates epithelial migration, granulation, and angiogenesis [29, 30]. However, existing hydrogel designs still face significant challenges in achieving multifunctional synergy and a balance of performance. Single‐component systems (e.g., pure fibrin or dextran gels) suffer from inherent drawbacks, including weak mechanical properties, uncontrolled degradation, and limited biofunctionality. Although physical blends or single‐network composites can partially optimize certain properties, they often fail to achieve temporally coordinated synergy among multiple functions, such as hemostasis, adhesion, anti‐inflammation, and orderly regeneration within the dynamic wound microenvironment. While dual‐network hydrogels have attracted considerable attention in recent years, research efforts have largely focused on synthetic polymer systems, with functionalization strategies that rely heavily on exogenous drug or bioactive factor loading [31]. This not only increases preparation complexity but also carries risks of burst release, inactivation, and potential immunogenicity. Natural polysaccharides, such as dextran, offer outstanding biocompatibility and chemical versatility over synthetic polymer‐based materials. Their abundant hydroxyl groups allow functionalization with methacrylate or thiol groups, enabling in situ crosslinking and injectability [32, 33, 34, 35]. Meanwhile, fibrin, a key component of the natural coagulation cascade, exhibits intrinsic hemostatic and pro‐angiogenic properties. However, fibrin hydrogels alone suffer from weak mechanics and rapid degradation, which limit their clinical utility [33, 36].

To address these limitations, we designed and fabricated an injectable dual‐network hydrogel integrating bioinspired adhesion, tunable mechanics, and multifunctional bioactivity. The hydrogel integrates fibrin with methacrylated dextran and catechol‐modified DMA. The design couples the hemostatic and cytocompatible nature of fibrin with the mechanical tunability and anti‐inflammatory capacity of dextran [37, 38, 39, 40, 41], while catechol groups confer robust tissue adhesion inspired by mussel foot proteins [42, 43, 44, 45, 46]. The hydrogel forms in situ through the dual crosslinking of fibrinogen and dextran‐MA–DMA, triggered by thrombin and dithiothreitol (DTT). Upon injection, the precursor solution forms a gel rapidly at 37°C, conforming to irregular wound geometries and forming a stable adhesive barrier. This bioinspired dual‐network structure achieves integrated hemostasis, anti‐inflammation, and regenerative modulation through multimechanistic synergy within the dynamic wound microenvironment. It simultaneously accomplishes rapid hemostasis, orderly inflammation resolution, and scar minimization in complex trauma repair. The “hemostasis‐anti‐inflammation‐regeneration” integrated progression design surpasses existing materials that typically focus on only a single stage or function. We hypothesized that this bioinspired dual‐network architecture would enable synergistic hemostatic and immunomodulatory effects, accelerate wound closure, and reduce scar formation. To validate this hypothesis, we systematically characterized the material properties of the dual‐network hydrogel, including microstructure, mechanical properties, adhesive strength, and ROS scavenging capability, and evaluated its in vitro hemostatic performance, cytocompatibility, and immunomodulatory functions. In rat liver and tail‐amputation models, this fibrin–dextran‐MA–DMA hydrogel reduced blood loss to 24% and shortened bleeding time to 18% compared with commercial gelatin sponges. RNA‐seq, immunofluorescence, and histological analysis revealed that the hydrogel promotes scarless wound healing by activating antioxidant defense pathways, remodeling the ECM, driving macrophage polarization toward an M2 phenotype, and enhancing angiogenesis [47, 48, 49, 50, 51]. Collectively, these findings demonstrate that integrating biological hemostasis with chemical crosslinking and catechol‐based adhesion can achieve rapid hemostasis, immunomodulation, and scar‐free tissue regeneration, offering a promising, generalizable strategy for complex and emergency wound management.

## 2 Results and Discussion

### 2.1 Preparation and Characterization of Hydrogels

**FIGURE 1:**
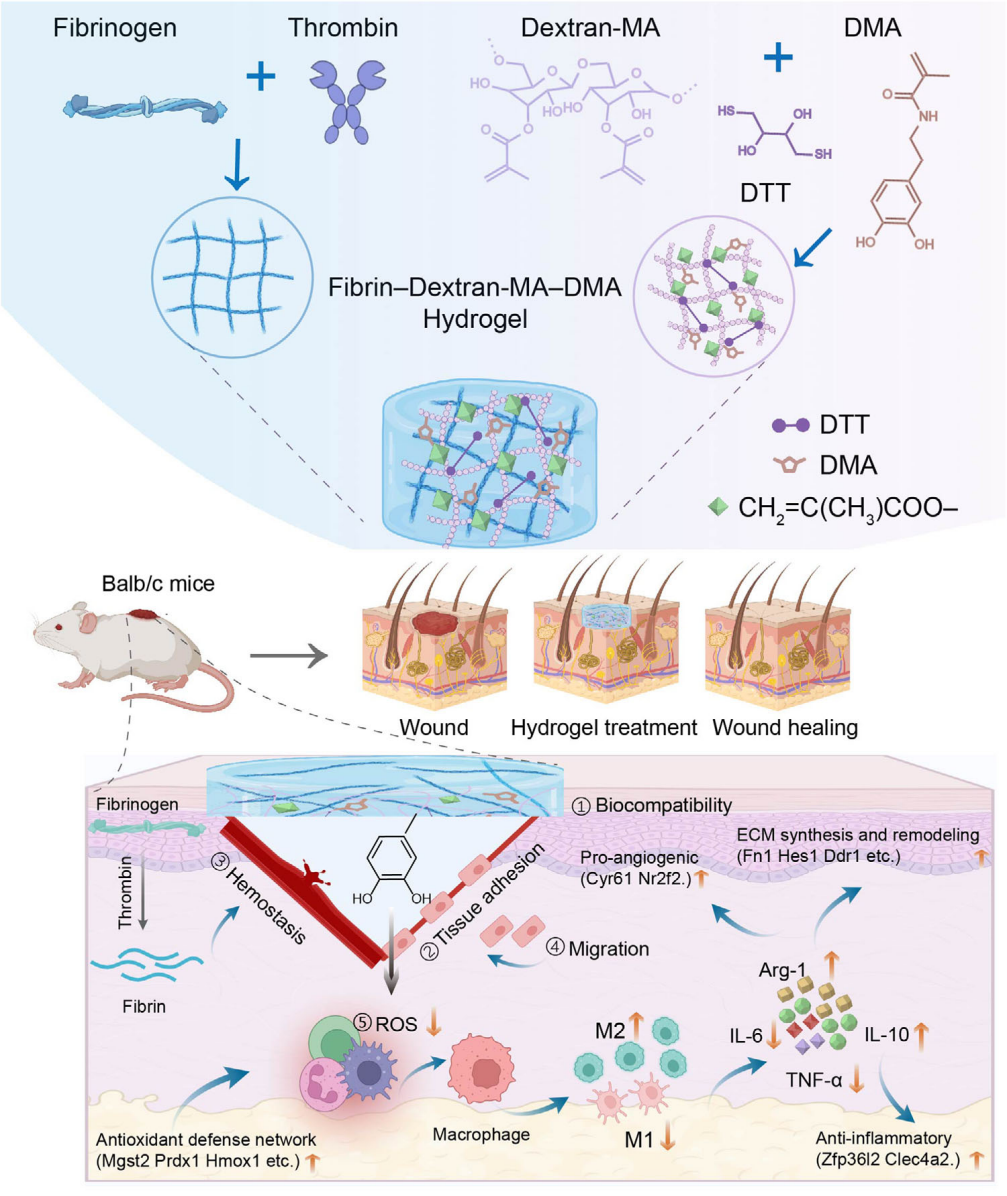
Schematic illustration of the synthesis of the fibrin–dextran‐MA–DMA hydrogel. Dual crosslinking of fibrinogen and dextran‐MA–DMA is achieved via thrombin‐catalyzed polymerization and DTT‐mediated thiol‐ene coupling, forming a cohesive double‐network structure. The drawing is a schematic representation and is not to scale. Image created with BioRender.com, with permission.

**FIGURE 2:**
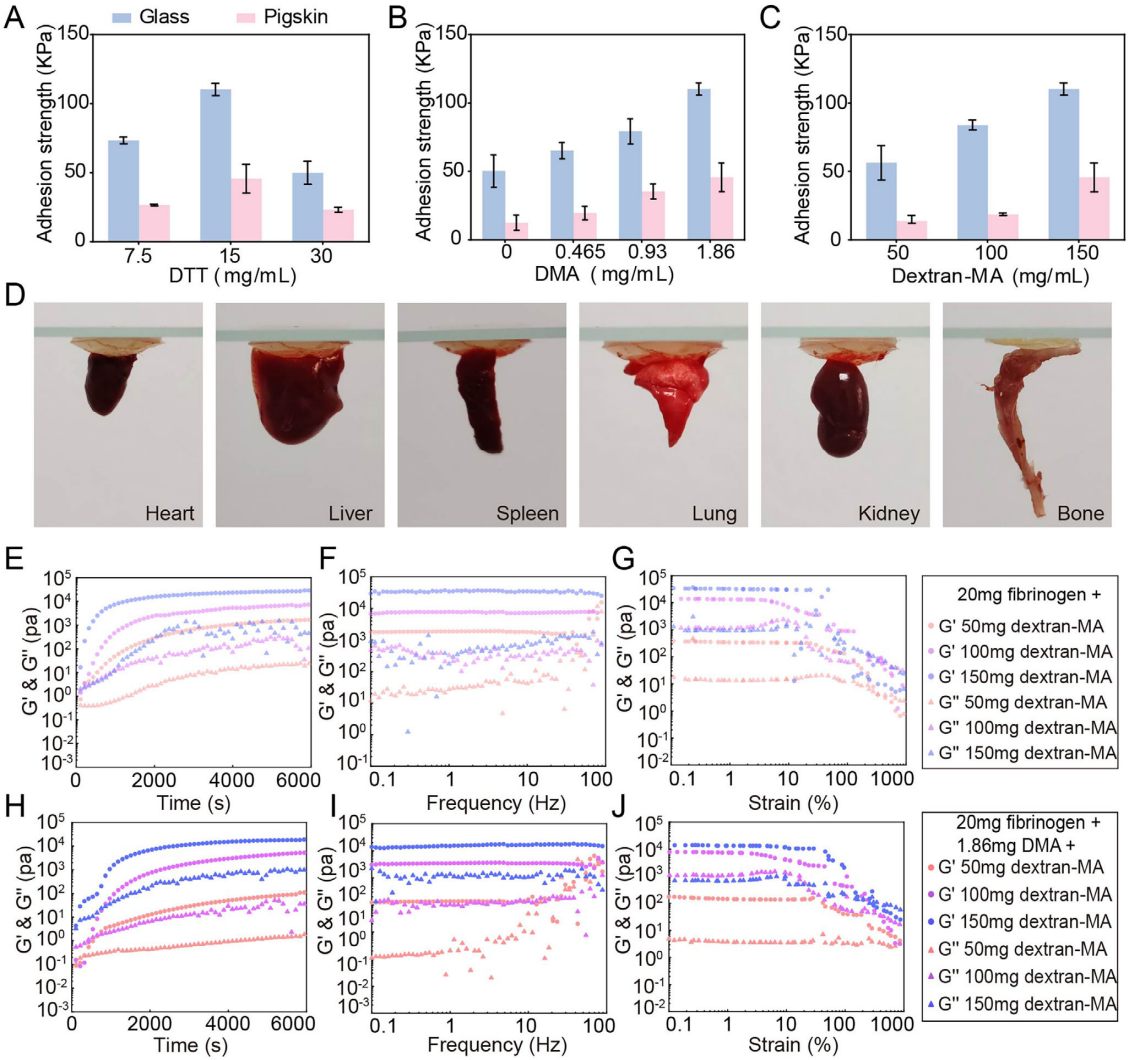
Adhesion performances of hydrogel samples with different concentrations of DTT, DMA, and dextran‐MA (A–D). Data are presented as mean ± SD (*n* = 3). *G*′ and *G*″ of different hydrogels with respect to oscillation time (E, H), frequency (F, I), and strain sweep (G, J). (E–J) fibrinogen (20 mg/mL), thrombin (20 U/mL).

We utilized fibrin and dextran‐methacrylate (dextran‐MA) (Figure S1) as the basic components, with DMA serving as the adhesive constituent for in situ gelation, to prepare a fibrin−dextran‐MA–DMA hydrogel (Figure 1). Thrombin was employed for the cleavage of fibrinogen, while DTT functioned as the cross‐linking agent. The mixture was injectable prior to gelation (Figure S2C). It was then incubated at 37°C for 1 h to ensure complete gelation (Figure S2A,B). As shown in Figure S2D, time‐sweep rheology at 37°C quantitatively confirms this process. The storage modulus (G′) exceeds the loss modulus (G″) at approximately 107 s, indicating the onset of gelation. The mechanical properties of these hydrogels were determined by rheometry. The adhesive properties of the hydrogel to different substrates were evaluated using the lap‐shear adhesion test [42, 44, 52, 53, 54]. The hydrogel demonstrated significantly enhanced adhesive capabilities to substrates such as glass and pigskin compared to the DMA‐free hydrogels. DMA and DTT differentially influenced adhesion. DMA content showed a strong positive correlation with adhesion strength (Figure 2B), consistent with its role as an adhesive moiety. In contrast, the effect of DTT on adhesion saturated beyond an optimal concentration (Figure 2A), as its primary function is to increase crosslinking density and mechanical integrity rather than provide interfacial binding. Furthermore, dextran‐MA content was a critical determinant of adhesion, defining the network's capacity to present functional groups (Figure 2C). The hydrogel demonstrated a maximum adhesion strength of 115.46 kPa when applied to glass substrates, compared to 38.17 kPa observed on porcine skin substrates (see Table S1 for the detailed ingredients of the hydrogel preparation). Furthermore, the hydrogel exhibited strong adhesion to various mouse organs, including heart, liver, spleen, lung, kidney, and bone (Figure 2D). To elucidate the intrinsic mechanism behind the robust, universal adhesion of this dual‐network hydrogel, we conducted an in‐depth analysis based on established catechol chemistry principles combined with our unique material design. The adhesive performance demonstrated by lap‐shear tests across various substrates can be attributed to the synergistic effect between interfacial molecular interactions and bulk network mechanics. The core of adhesion lies in the dopamine methacrylamide units, whose catechol groups form multiple chemical bond types with substrate surfaces, a principle inspired by marine mussel adhesion. We propose that catechol groups participate in the following interactions: coordination bonds with metal ions or oxides on inorganic surfaces; covalent bonds with amine/thiol groups abundant in biological tissues; and hydrogen bonds, as well as *π*–*π* and cation‐*π* interactions with diverse organic and inorganic surfaces. This versatile, multimodal bonding enables the hydrogel to adapt to form strong connections with chemically diverse substrates. These results indicate its potential application in a wide range of clinical scenarios.

The rheological characteristics of the hydrogel samples were measured to evaluate the stability and viscoelastic properties of their internal structures. During the oscillatory time sweep experiments (Figure 2E), all hydrogels underwent gelation within minutes, and it was observed that the storage moduli (*G*′) of all hydrogel samples remained consistently higher than the loss moduli (*G*″) after gelation, which is indicative of the stability of the hydrogels. In the oscillatory frequency sweep (Figure 2F), smooth curves over the frequency range of 0.1–100 Hz were observed, highlighting the well‐maintained hydrogel structures and their elastomeric properties. The oscillatory strain sweep revealed that each sample demonstrated an intersection point between *G*′ and *G*″ within a strain range of 100%–1000% (Figure 2G). Before this crossover point, a linear viscoelastic region was evident, which indicated a stable internal structure, satisfactory viscoelasticity, and resistance to deformation. The hydrogel displayed an ultimate strain approaching 1000%, significantly exceeding the maximal strain capacity of human skin [55], thus confirming its exceptional viscoelastic behavior. It is noteworthy that the addition of DMA led to a reduction in both *G*′ and *G*″ across all oscillatory time, frequency, and strain sweeps (Figure 2H–J). This phenomenon is attributed to the slight increase in water content resulting from the incorporation of the hydrophilic DMA. Rheological time‐sweep data (Figure 2H) show that the storage modulus increases with rising monomer concentration: from 116.93 Pa (Fib_20_D_50_) to 18.19 KPa (Fib_20_D_150_). Amplitude‐sweep data (Figure 2I) further indicate that gels formed from higher‐concentration solutions exhibit greater structural strength and rigidity. These results demonstrate, from a mechanical perspective, that higher monomer concentrations yield stiffer and tougher polymer networks.

The swelling ratio of the hydrogels was quantitatively assessed by immersing them in PBS for 24 h. As shown in Figure S3, the equilibrium swelling ratio decreases with increasing monomer concentration: from 711.33% ± 14.90% (Fib_20_D_50_) to 271.85% ± 15.12% (Fib_20_D_150_). According to the Flory–Rehner theory, the equilibrium swelling ratio is inversely proportional to the crosslinking density. The decrease in hydrogel swelling with increasing dextran‐MA concentration can be attributed to the formation of a denser crosslinked structure at higher monomer concentrations. As shown in Figure S4, with increasing dextran‐MA monomer concentration from D_50_ to D_150_, the hydrogel microstructure undergoes systematic changes: D_50_ displays a microporous network structure with relatively thin pore walls. D_150_ evolves into a homogeneous morphology with significantly reduced pore size, thickened pore walls, and a highly dense network. This microstructural evolution from “loose” to “dense” provides direct morphological evidence for increased polymer network crosslinking and reduced mesh size.

### 2.2 Biocompatibility and Degradation Characteristics of Hydrogels

**FIGURE 3:**
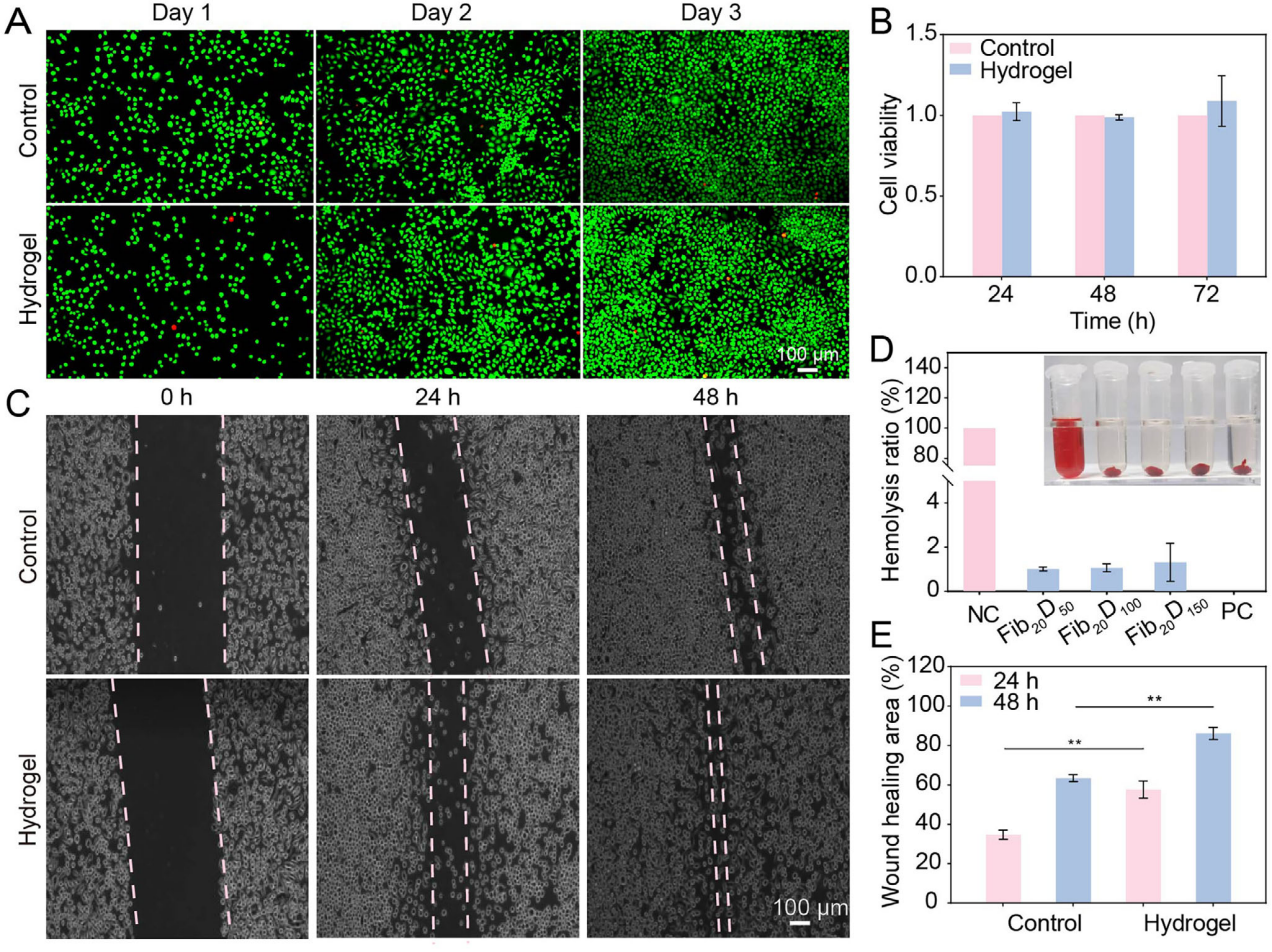
In vitro cytocompatibility and hemocompatibility properties of hydrogels. (A) Live/Dead staining of L929 cells after incubation with hydrogels for 24, 48, and 72 h. The scale bar is 100 µm. (B) CCK‐8 assay (*n* = 3). (C) Cell migration experiment of L929 cells for 24 and 48 h. The scale bar is 100 µm. (D) Hemolysis test of hydrogels. Fib_20_D_50_ (fibrinogen 20 mg/mL, dextran‐MA 50 mg/mL, DTT 5 mg/mL, DMA 1.86 mg/mL, thrombin 20 U/mL), Fib_20_D_100_ (fibrinogen 20 mg/mL, dextran‐MA 100 mg/mL, DTT 10 mg/mL, DMA 1.86 mg/mL, thrombin 20 U/mL), Fib_20_D_150_ (fibrinogen 20 mg/mL, dextran‐MA 150 mg/mL, DTT 15 mg/mL, DMA 1.86 mg/mL, thrombin 20 U/mL) (*n* = 6). (E) Cell migration rate statistics (*n* = 3). Data are presented as mean ± SD. Statistical significance was assessed using Student's *t*‐tests; ^**^ *p* < 0.01.

Cytocompatibility, hemocompatibility, and histocompatibility are essential prerequisites for biomedical applications and are of great importance in biomaterials research. The cytotoxicity of the hydrogels was assessed using a series of in vitro assays. Proliferation activity of L929 fibroblasts was evaluated via LIVE/DEAD staining, indicating that the majority of cells retained viability, as evidenced by green fluorescence, and were capable of proliferating (Figure 3A). Cell viability was further quantitatively assessed using the CCK‐8 assay after incubating the L929 cells for 24, 48, and 72 h (Figure 3B). These results confirmed that the hydrogels exhibited excellent cytocompatibility. In addition, hydrogels prepared with different monomer concentrations successfully passed the hemolysis test, with a hemolysis ratio below 2% (Figure 3D), meeting the ISO 10993–4 standard, which stipulates a hemolysis ratio of less than 5%.

In the application of hydrogels as wound dressings, it is essential that the hydrogel exhibits properties that promote cell migration, thereby facilitating accelerated wound healing. In the cell migration assay (Figure 3C), L929 cells exhibited directional movement toward the unoccupied regions over time. Quantitative analysis revealed that the migration rate stimulated by the hydrogel extract reached 86.15% ± 3.10% at 48 h, a statistically significant increase compared to the control group, which demonstrated a migration rate of 57.70% ± 4.34% (Figure 3E). This observation was corroborated through the replication of the experiment using mouse umbilical vein endothelial cells (MUVECs) (Figure S5). The enhanced cellular adhesion and migration are attributed to the presence of the RGD (arginine‐glycine‐aspartic acid) sequence within the fibrin α chain, which enhances cell adhesion and migration by binding to integrins [56, 57, 58].

**FIGURE 4:**
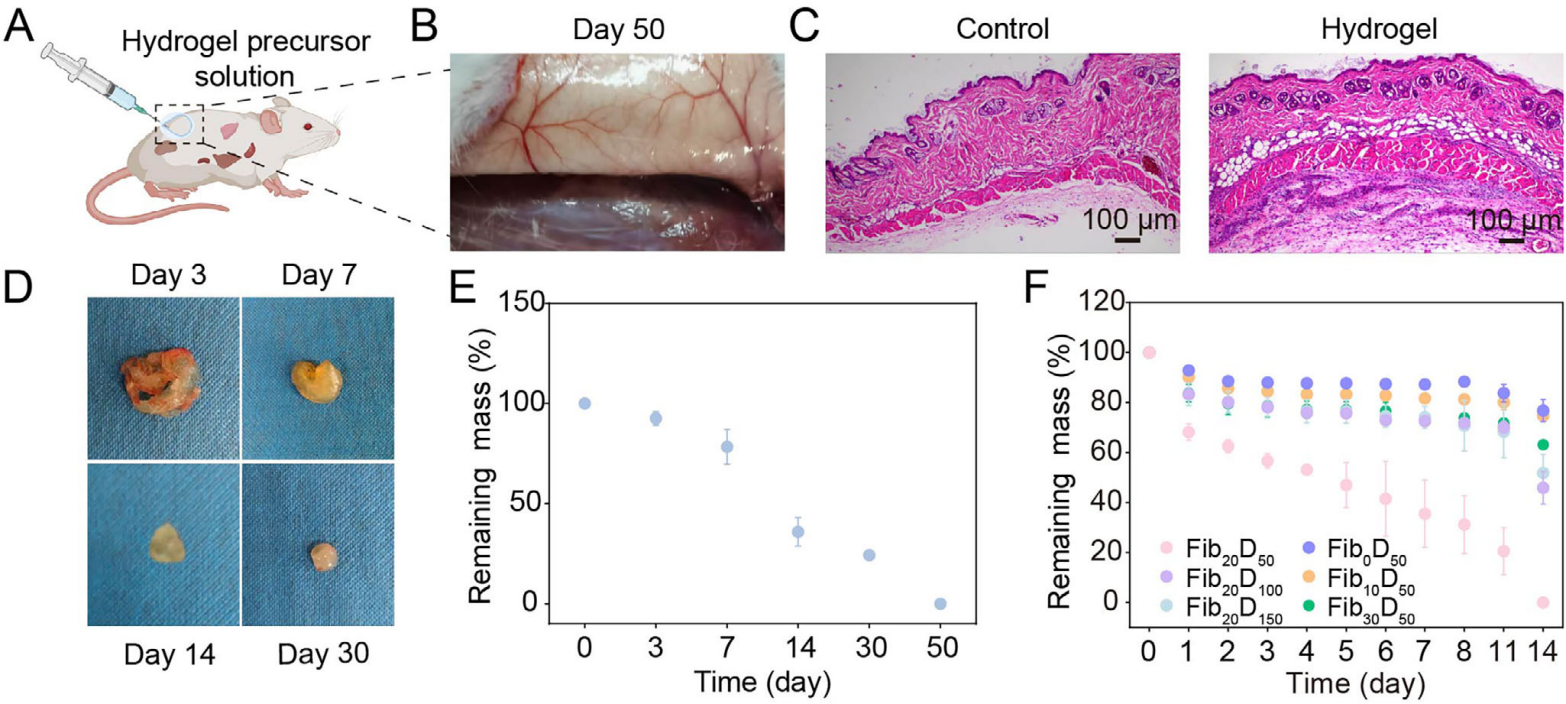
(A, B) In vivo degradation and histocompatibility properties of hydrogels. Figure A was created with BioRender.com, with permission. (C) Hematoxylin and Eosin (H&E) staining results of the host tissue surrounding the degrading hydrogel. The scale bar is 100 µm. (D) Volume change of hydrogels during in vivo degradation. (E) Mass loss of hydrogels during in vivo degradation (*n* = 3). (F) In vitro degradation of hydrogels (*n* = 3). Fib_0_D_150_ (dextran‐MA 150 mg/mL, DTT 15 mg/mL, DMA 1.86 mg/mL), Fib_10_D_150_ (fibrinogen 10 mg/mL, dextran‐MA 150 mg/mL, DTT 15 mg/mL, DMA 1.86 mg/mL, thrombin 10 U/mL), Fib_30_D_150_ (fibrinogen 30 mg/mL, dextran‐MA 150 mg/mL, DTT 15 mg/mL, DMA 1.86 mg/mL, thrombin 30 U/mL). Data are presented as mean ± SD.

The degradation properties of hydrogels are of great importance in tissue engineering and regenerative medicine. To evaluate this, we synthesized cylindrical hydrogel samples, maintaining identical volume while varying their composition. It was observed that the degradation rate increased as the dextran‐MA concentration decreased (Figure 4F). Specifically, the Fib_20_D_50_ formulation completely degraded within 14 days, whereas the Fib_20_D_150_ formulation retained 76.83 ± 3.67% of its mass over the same duration. Consequently, the degradation kinetics of the fibrin–dextran‐MA–DMA hydrogels can be effectively modulated by adjusting the dextran‐MA content and the cross‐linking. Based on its optimal balance of mechanical strength, adhesive properties, and in vitro degradation kinetics, the Fib_20_D_150_ hydrogel was chosen for further evaluation in both in vitro and in vivo models.

To further elucidate the degradation mechanism of the fibrin–dextran‐MA–DMA hydrogel, its degradation products were characterized using FTIR and ^1^H NMR spectroscopy, and the corresponding results are shown in Figure S6. FTIR analysis revealed a significant attenuation of the characteristic peaks at 1736 cm^−^^1^ (assigned to the C═O stretching vibration of DMA ester bonds) and 1654/1546 cm^−^^1^ (corresponding to the amide I/II bands of fibrin), which indicated the hydrolytic cleavage of both DMA moieties and the proteinaceous fibrin component within the hydrogel network. Meanwhile, the broad O–H stretching band at 3415 cm^−^^1^ remained prominent, which was consistent with the preserved dextran backbone and the hydroxyl end‐groups generated during the degradation process. ^1^H NMR spectroscopy provided complementary structural insights into the degradation products. The characteristic signals in the *δ* 3.0–3.8 ppm range were assigned to the dextran backbone, demonstrating the partial retention of the polysaccharide skeleton after degradation. Aromatic proton signals at *δ* 6.6–6.8 ppm confirmed that the phenyl ring moieties of DMA existed as inert fragments in the degradation products, while the absence of vinyl proton signals at *δ* 5.0–6.5 ppm suggested the complete consumption of DMA double bonds during hydrogel network formation. Additionally, the distinct signals at *δ* 1.76 ppm and *δ* 5.4 ppm were attributed to DTT, confirming its presence in the degradation system; the other proton signals of DTT overlapped with the background signals in the *δ* 2.5–4.0 ppm range and were thus not distinguishable. Notably, no clear amide proton signals were detected in the *δ* 6.5–8.0 ppm region, which was in good agreement with the FTIR results confirming the cleavage of fibrin amide bonds. Nevertheless, the FTIR and ^1^H NMR data clearly demonstrate that the hydrogel degradation proceeds predominantly via the selective hydrolysis of labile ester and amide bonds. In contrast, the rigid dextran backbone and DMA phenyl ring fragments are retained as residual structural moieties. This observation confirms that the hydrogel undergoes bulk erosion at the labile crosslinking sites, rather than degrading into small monomeric molecules.

Histocompatibility is another critical criterion for evaluating biocompatibility, reflecting the host response to implanted hydrogels. To investigate the biodegradability and histocompatibility of fibrin–dextran‐MA–DMA hydrogels, the F_20_D_150_ hydrogel was injected into the dorsal region of mice (Figure 4A). Following implantation, the hydrogels exhibited a progressive reduction in volume, thereby confirming their biodegradability in vivo (Figure 4D). As shown in Figure 4E, the remaining mass (%) of fibrin–dextran‐MA–DMA hydrogels was measured. After 30 days, the residual mass was 24.30% ± 2.25%, and complete degradation occurred by day 50. Importantly, no indications of necrosis or infection were observed in the adjacent muscle or dermal layers surrounding the injection site (Figure 4B). Histological assessment of peri‐implant tissues via Hematoxylin and Eosin (H&E) staining revealed that the fibrin–dextran‐MA–DMA hydrogels did not provoke significant inflammatory responses in the surrounding tissues (Figure 4C). After subcutaneous injection of the hydrogel, as the hydrogel underwent degradation, new connective tissue formed around the hydrogel due to the recruitment of fibroblasts, rather than the formation of a capsule layer. This indicates a synchronous process of new tissue formation and hydrogel degradation. Notably, a well‐aligned, newly formed collagen‐rich tissue was observed at the hydrogel‐tissue interface, indicating the material's potential to promote wound healing processes. Hydrogel degradation results indicated that the hydrogel degradation was faster in open wounds than in subcutaneous (residual mass at day 7: 47% ± 6.08% vs. 78.36% ± 8.7%) (Figure S7). This difference is attributable to higher proteolytic enzyme activity, continuous exudate exchange, and more active cell‑mediated clearance in the wound environment. Furthermore, no significant pathological changes were observed in major organs, such as the heart, liver, spleen, lungs, or kidneys (Figure S8). Overall, both in vitro and in vivo evaluations have demonstrated that the hydrogel exhibits excellent biocompatibility and is sufficiently safe for further in vivo investigations.

### 2.3 In Vitro and In Vivo Hemostatic Performance of the Hydrogels

**FIGURE 5:**
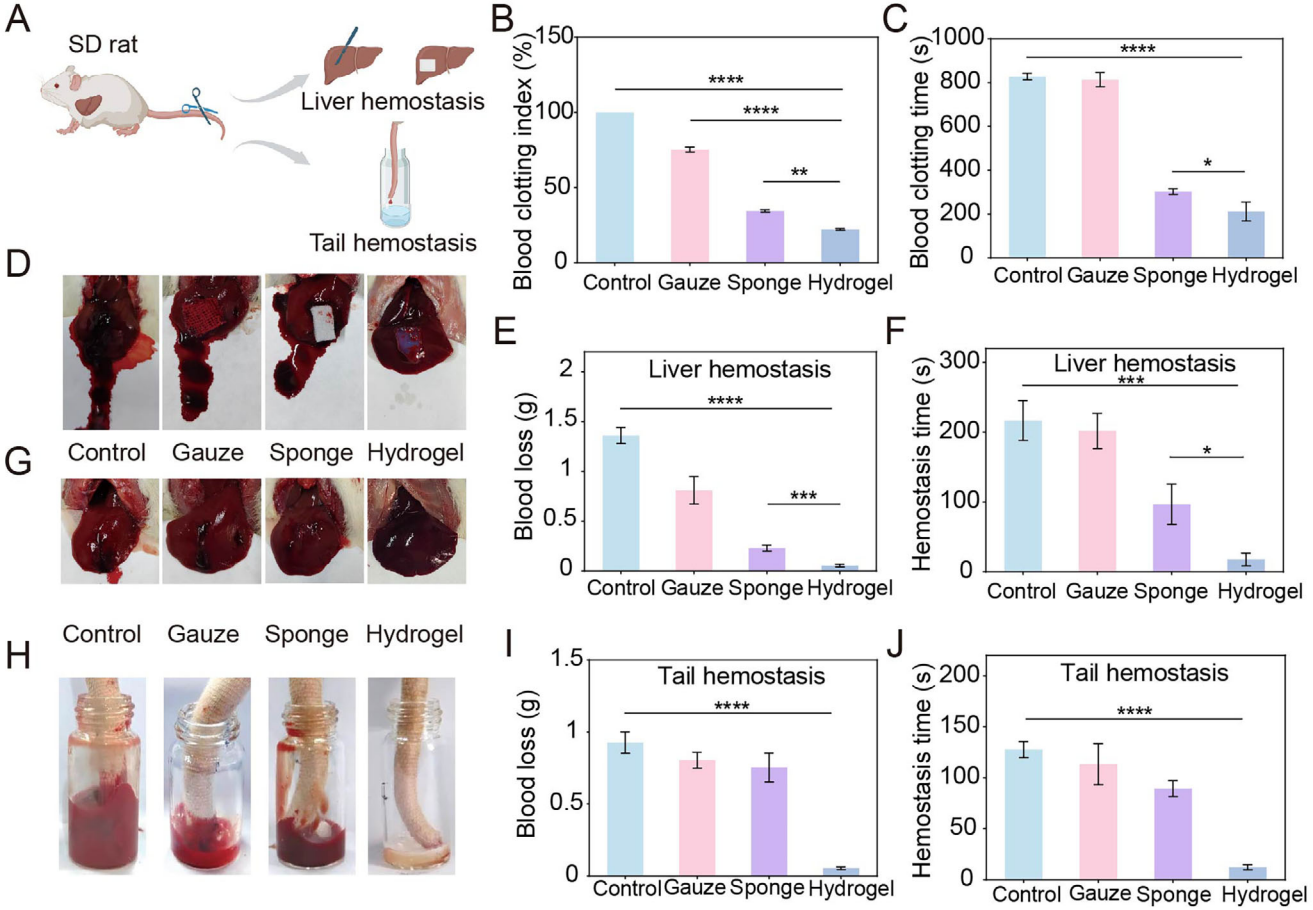
In vitro and in vivo hemostasis performances of the hydrogels. (A) Schematic of a rat liver and tail incision hemostasis model. Image created with BioRender.com, with permission. (B) Blood Clotting Index. (C) Whole blood coagulation time. (D–F) Images and data statistics of the hemostasis process in a rat liver following a 10 mm long and 2 mm deep incision, comparing gauze, sponge, and hydrogel treatments. (G) After the dressing was removed from the liver. (H–J) Images and data statistics of the hemostasis in a rat tail, comparing gauze, sponge, and hydrogel treatments. Data are presented as mean ± SD (*n* = 3). Statistical significance was assessed using Student's *t*‐tests and one‐way ANOVA; ^*^ *p* < 0.05, ^**^ *p* < 0.01, ^***^ *p* < 0.001, and ^****^ *p* < 0.0001.

The in vitro pro‐coagulant activity of the hydrogel was assessed by measuring the Whole Blood Coagulation Index (BCI). The comparative analysis of the hemostatic efficacy of the hydrogel was conducted against medical gauze and hemostatic sponges. As shown in Figure S9, the gauze group resembled the blank control group, exhibiting a bright red color indicative of non‐coagulated blood. Upon the addition of distilled water, hemolysis occurred, resulting in the rupture of red blood cells. In contrast, the upper layer of the sponge group was pale red, indicating partial coagulation, whereas the upper layer of the hydrogel group was clearly transparent. Quantitative analysis of BCI values for the different materials was performed (Figure 5B). While gauze and sponge are widely used as hemostatic agents in clinical practice, neither of these materials show significant blood coagulation performance. The BCI recorded for gauze was 75.30% ± 1.61%, for sponge 34.56% ± 0.88%, whereas the hydrogel demonstrated a significantly lower BCI of 22.20% ± 0.60%.

In Figure 5C, a comparative analysis reveals that hydrogel‐induced blood clot formation occurs at a significantly faster rate (302.7 ± 12.86 s) than in the blank group (827.3 ± 15.01 s) and the gauze group (813.7 ± 32.04 s). This phenomenon can be attributed to the presence of thrombin within the hydrogel, which cleaves fibrinogen and forms a fibrin network. This densely packed network structure mechanically captures red blood cells and platelets, thereby impeding blood flow. Additionally, the α‐chain of fibrin contains RGD, which has the capability to bind to the platelet surface integrin (GPIIb/IIIa) [57, 58]. This interaction triggers platelet degranulation and the subsequent release of procoagulant factors, such as ADP and TXA_2_, effectively speeding up the coagulation process. Moreover, the catechol groups present in DMA have been shown to enhance the adhesion and activation of blood cells, consequently improving their clotting efficacy [59, 60, 61].

The in vivo hemostatic efficacy of the hydrogel was initially demonstrated by its coagulation properties in vitro and subsequently verified using animal models of hemostasis (Figure 5A). In the rat liver hemorrhage model, the hydrogel group exhibited a significantly smaller blood diffusion area compared to the other groups (Figure 5D). As shown in Figure 5E,F, in the control group, the rat required 216.7 ± 28.45 s of hemostasis time and experienced a blood loss of 1361 ± 81 mg. In contrast, the hydrogel group achieved hemostasis in 17.7 ± 9.3 s with 56.7 ± 13.7 mg of blood loss, significantly outperforming the commercial medical sponge, which required 96.7 ± 28.9 s and resulted in 232.7 ± 30.0 mg of blood loss. Upon removal of the dressing, the liver surface was examined for residual bleeding. No bleeding was observed 5 min after the hydrogel was removed, whereas trace blood extravasation was observed at sites covered with gauze or the commercial hemostatic sponge (Figure 5G). This confirmed the pro‐coagulant properties of the hydrogel dressing. The hemostatic capacity was consistently demonstrated in the rat tail transection model, where the hydrogel achieved complete hemostasis within 12.33 ± 1.78 s, mirroring previous findings in liver injury models (Figure 5H–J). To compare with previous hemostatic materials containing similar components, we used fibrin glue (which shares the same biological components as the material used in this study) and dextran‐based hydrogel as controls (Figure S10). The results demonstrated that, compared with the fibrin groups, the dual‐network hydrogel significantly shortened bleeding time in both SD rat liver hemorrhage and tail amputation models (from 96.43 to 17.67 s, and from 66.33 to 12.33 s, respectively) and reduced the blood loss (from 422 to 54.33 mg, and from 254 to 56.67 mg, respectively). These findings confirm the synergistic enhancement of hemostatic efficacy conferred by the dual‐network structure, which integrates rapid fibrin clotting with robust mechanical support from the dextran‐based network. These results indicate that the hydrogel exhibits superior wound‐sealing and procoagulant properties, which can be attributed to the activation of the coagulation cascade and enhanced thrombus formation due to the presence of fibrin and thrombin in the hydrogel. Moreover, the hydrogel allows for safe detachment without causing secondary tissue injury and achieves effective hemostasis with minimal or no applied pressure, making it particularly suitable for wounds on visceral organs and a promising candidate for clinical application [39].

### 2.4 In Vitro Immunomodulatory Mechanism

Excessive production of ROS affects normal physiological metabolic processes and subsequently hinders wound healing. The antioxidant efficacy of the hydrogel and its constituent components was assessed using DPPH and PTIO, which are stable nitrogen‐ and oxygen‐centered radicals, respectively. As shown in Figure S11B,C, both the hydrogel and its individual components demonstrated significant ROS‐scavenging activity compared to the control group, achieving near‐quantitative scavenging of DPPH and PTIO radicals. This agrees with previous findings of robust free‐radical‐scavenging performance and was visually confirmed by the marked fading of the DPPH and PTIO solutions [47]. These results indicate that the hydrogel exhibits exceptional antioxidant capacity for ROS elimination and is highly promising for applications in wound healing. The ROS‐scavenging ability of the hydrogel was evaluated using L929 cells. DCFH‐DA was utilized as a fluorescent probe to quantify intracellular ROS after co‐culture with hydrogel leachate, the probe emits fluorescence upon oxidation by ROS, thereby serving as an indicator of ROS levels and the material's scavenging efficacy. As shown in Figure S11A, the fluorescence intensity significantly decreased in cells cultured with fibrin and dextran. In contrast, the hydrogel group exhibited almost complete absence of fluorescence, suggesting that the hydrogel played a crucial role in ROS scavenging. This observation is consistent with the in vitro ROS‐scavenging results (Figure S11D). The superior antioxidant activity is attributed to the catechol groups of DMA and the abundant reducing hydroxyl groups of dextran. Collectively, these findings suggest that the hydrogel can accelerate wound healing by effectively eliminating excess ROS.

**FIGURE 6:**
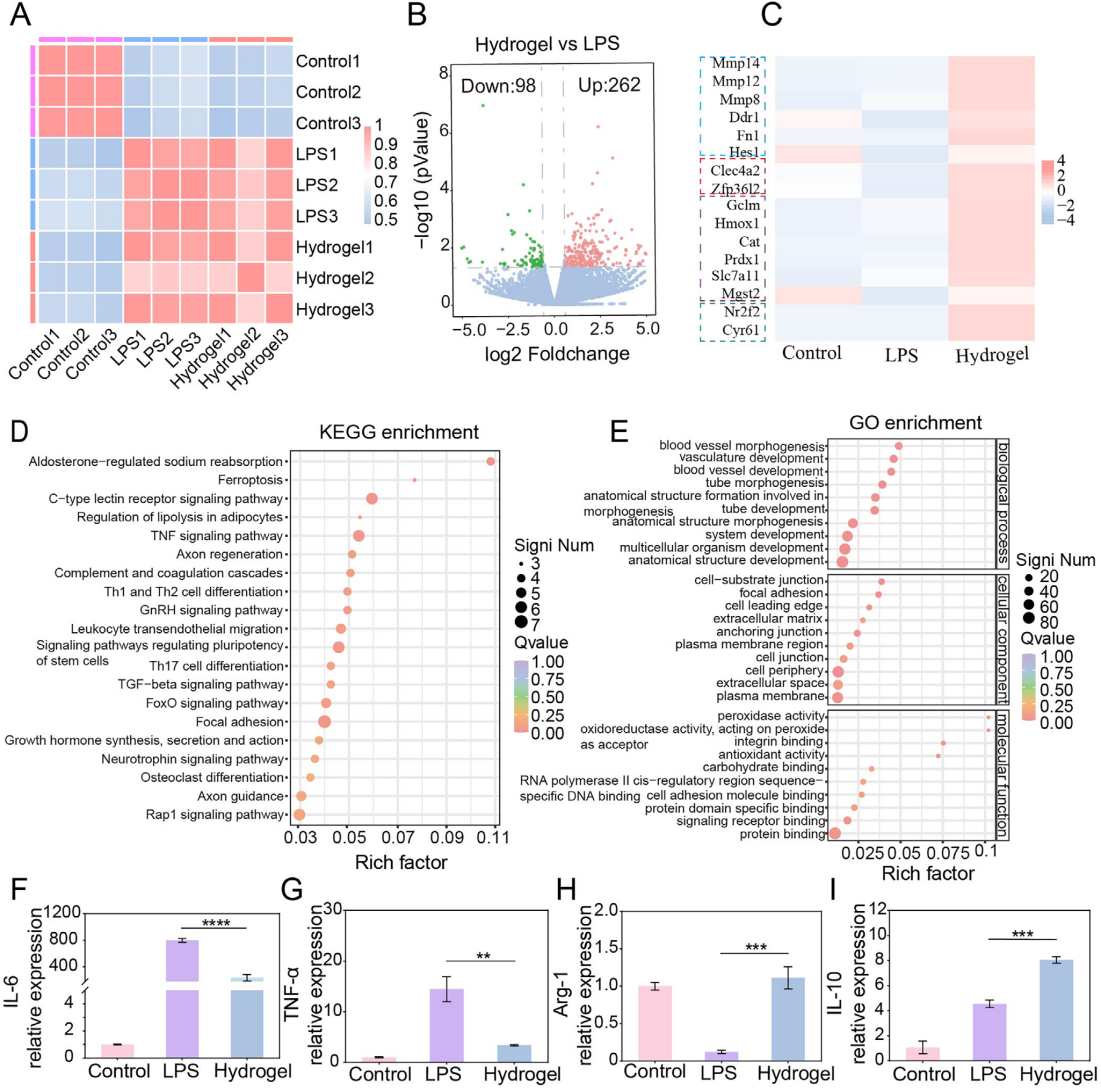
(A) Heatmap of inter‐sample correlation analysis. (B) Volcano plot of DEGs. (C) Clustering heatmap of the expression patterns of key DEGs. (D) KEGG pathway enrichment analysis of DEGs. (E) Scatter plot of significantly enriched Gene Ontology (GO) terms. (F‐I) mRNA expression levels of IL‐6, TNF‐α, Arg‐1 and IL‐10 (*n* = 3). Data are presented as mean ± SD. Statistical significance was assessed using Student's *t*‐tests; ^**^ *p* < 0.01, ^***^ *p* < 0.001, and ^****^ *p* < 0.0001.

To elucidate the anti‐inflammatory mechanism underlying macrophage treatment with the hydrogel, RNA‐seq was conducted on RAW 264.7 cells. These macrophages were polarized to the M1 phenotype using lipopolysaccharide (LPS) to mimic an inflammatory microenvironment, followed by a comparative analysis of gene expression profiles among cells treated with PBS, LPS, or LPS with the hydrogel. Pearson correlation analysis confirmed high data quality and reproducibility across biological replicates (Figure 6A, increasingly red indicates higher correlation) [47, 62]. Differential expression analysis identified 360 genes exhibiting significant expression changes in hydrogel‐treated RAW 264.7 cells compared with the LPS group, with 262 upregulated and 98 downregulated (Figure 6B). Hierarchical clustering of the representative differentially expressed genes (DEGs) identified four distinct gene‐expression modules associated with critical stages of wound healing (Figure 6C).

The first gene‐expression module (blue) shows that the hydrogel promotes ECM synthesis and remodeling. The upregulation of Fn1 enhances ECM formation [63, 64]. The downregulation of Hes1 expression inhibits the differentiation of epithelial stem cells into myofibroblasts, thereby reducing scar tissue formation [65]. Simultaneously, the discoidin‐domain receptor DDR1 activates matrix metalloproteinases (MMPs) that locally degrade ECM, thus creating space for cellular movement, proliferation, and new matrix deposition [66]. The second module (red) demonstrates that the hydrogel positively modulates inflammatory responses. By upregulating Zfp36l2, the hydrogel facilitates the targeted degradation of mRNAs encoding pro‐inflammatory cytokines (e.g., TNF‐α and IL‐6) and chemokines, thereby inhibiting the production of these mediators at the source [67]. Concurrently, the upregulation of Clec4a2 suppresses excessive inflammation [68]. The third module (purple) indicates that the hydrogel establishes a comprehensive antioxidant defense network. Slc7a11 imports cysteine, a precursor for the synthesis of glutathione (GSH), while Gclm catalyzes the rate‐limiting step in GSH production [69, 70]. The newly synthesized GSH is utilized by GST‐family enzymes such as Mgst2 to neutralize and detoxify harmful products formed during oxidative stress. Cat and Prdx1 directly scavenge ROS such as H_2_O_2_ [71, 72]. Hmox1 degrades heme to produce the antioxidant biliverdin and the cytoprotective signaling molecule carbon monoxide (CO), collectively mitigating oxidative damage, reducing inflammation, and promoting tissue repair [72, 73]. The fourth module (green) illustrates the pro‐angiogenic activity of the hydrogel. Cyr61 initiates angiogenesis by stimulating robust endothelial‐cell migration and proliferation, resulting in a preliminary, dense vascular network. Conversely, Nr2f2 moderates excessive endothelial proliferation and migration while strengthening inter‐endothelial junctions, thereby promoting vessel maturation. The coordinated upregulation of both genes is essential for the development of an abundant and functional neovasculature [40, 74, 75]. These transcriptional signatures were functionally validated in subsequent full‐thickness murine skin‐wound healing experiments.

Subsequent analyses utilizing Gene Ontology (GO) terms demonstrated that DEGs in both the LPS and hydrogel cohorts were associated with biological processes (BP), cellular components (CC), and molecular functions (MF) (Figure S12). Kyoto Encyclopedia of Genes and Genomes (KEGG) and GO enrichment analysis revealed that the hydrogel orchestrates a temporally coordinated response network that includes: (i) complement and coagulation cascades, which initiate immunomodulation, hemostasis, and tissue remodeling; (ii) aldosterone‐regulated sodium reabsorption, crucial for the maintenance of fluid and electrolyte homeostasis; (iii) Th1/Th2 and Th17 cell differentiation, driving immunomodulation and tissue repair; (iv) cell‐substrate junctions, focal adhesions, and integrin binding, which govern cell migration, tissue contraction, and structural remodeling; (v) blood vessel morphogenesis, which promote angiogenesis; and (vi) peroxidase, oxidoreductase, and antioxidant activities that are integral in the removal of H_2_O_2_ and the neutralization of ROS (Figure 6D,E). These results support a multimodal mechanism by which the hydrogel facilitates wound repair through ROS scavenging, reshaping of the immune microenvironment, and promotion of angiogenesis and tissue regeneration [76]. Further bar‐chart analysis (Figure 6F–I) shows that the hydrogel induces macrophage polarization from the M1 to the M2 phenotype by up‐regulating the expression of anti‐inflammatory mediators while down‐regulating pro‐inflammatory factors.

### 2.5 Wound Healing and In Vivo Anti‐Inflammatory Properties

**FIGURE 7:**
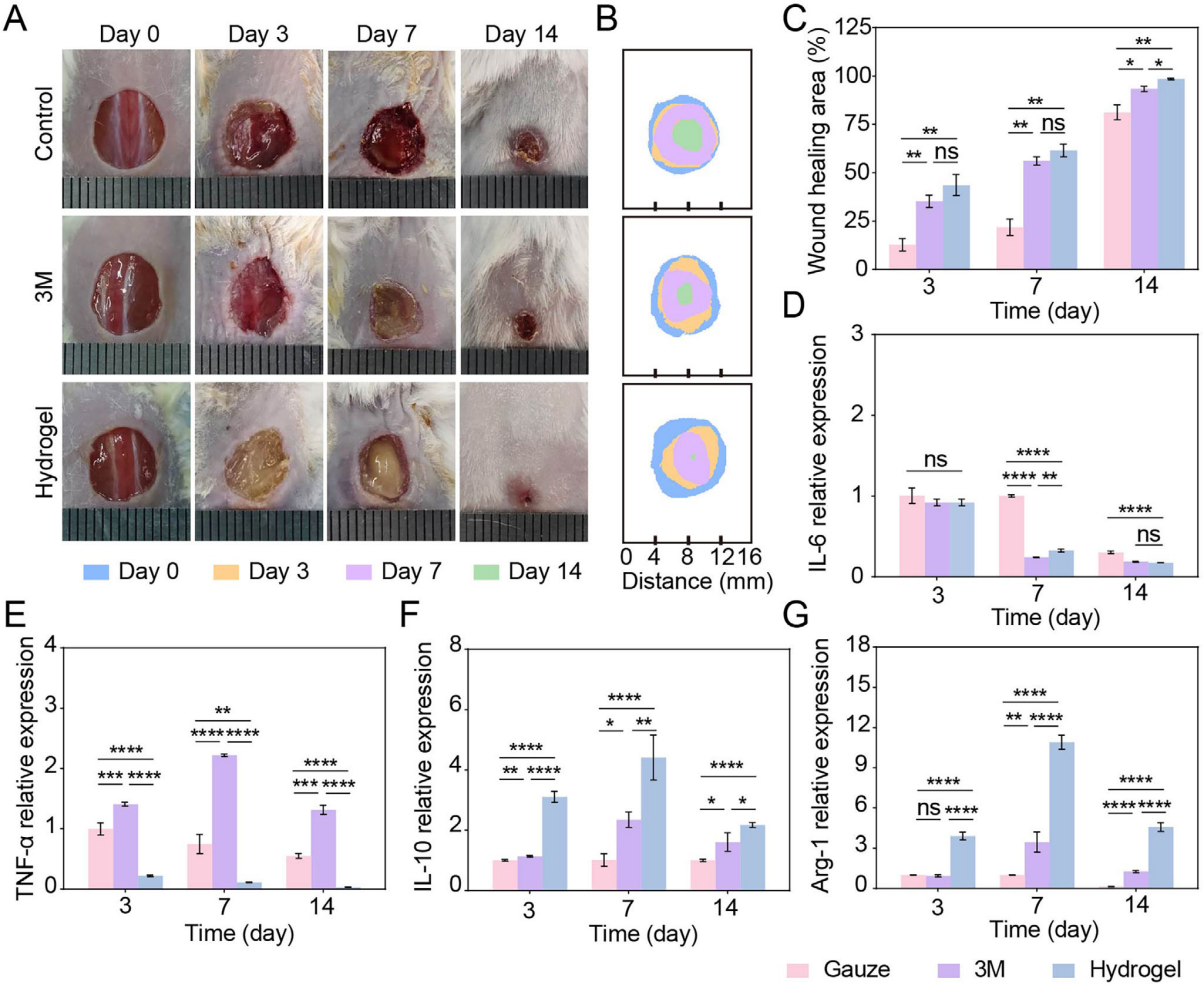
In vivo assessment of wound healing. (A) Representative images of wounds treated with gauze, 3 M, and hydrogel on days 0, 3, 7, and 14. (B) Schematic diagram of wound closure. (C) Wound healing rates on days 3, 7, and 14 (*n* = 5). (D–G) mRNA expression levels of IL‐6, TNF‐α, IL‐10, and Arg‐1 of wound tissues detected by qRT‐PCR (*n* = 3). Data are presented as mean ± SD. Statistical significance was assessed using Student's *t*‐tests and one‐way ANOVA; ns, not significant, ^*^ *p* < 0.05, ^**^ *p* < 0.01, ^***^ *p* < 0.001, and ^****^ *p* < 0.0001.

We conducted a comprehensive investigation into the efficacy of the hydrogel in promoting wound healing by employing a mouse full‐thickness skin wound model (Figure S13). In the control group, the wounds were treated with either commercial gauze or medical 3 M wound dressings, along with adhesive tape. In the experimental group, wounds were treated with hydrogel and adhesive tape. The wound healing process was monitored on days 0, 3, 7, and 14 (Figure 7A). The wound areas were measured using image analysis software to determine healing rates. Day 0 represents the initiation point at which full‐thickness skin wounds were inflicted and subsequently treated with different dressings. To facilitate visual analysis of healing rates across different groups, all time point wound margins were shown in Figure 7B, and healing rates were statistically analyzed (Figure 7C). By day 3, the wound dimensions in the control group exhibited negligible variation, whereas those treated with the hydrogel demonstrated a reduction in size. By day 7, partial healing was observed in all wounds, but with varying degrees of progress. By day 14, hydrogel‐treated wounds had achieved near‐complete closure, in contrast to the partially open wounds in both the gauze and 3 M groups. The hydrogel group consistently demonstrated the most rapid closure, achieving 98.47% ± 0.84% closure by day 14, compared with 81.21% ± 8.57% closure in the gauze group. This enhanced healing efficacy is attributed to the robust anti‐inflammatory characteristics of the hydrogel. Additionally, the moist wound environment facilitates the infiltration of polymorphonuclear leukocytes, thereby enhancing infection control—an intrinsic advantage of hydrogel dressings over conventional dry dressings.

Physiological levels of inflammatory factors promote wound healing. Nonetheless, an excessive inflammatory response often precipitates chronic inflammation, which in turn hinders tissue regeneration, leads to scar formation, and detrimentally affects skin functionality. Thus, expediting the progression from the inflammatory phase to the proliferative phase is paramount for achieving optimal wound repair. To further study the anti‐inflammatory efficacy of the hydrogel, quantitative reverse transcription‐polymerase chain reaction (qRT‐PCR) and immunofluorescence staining were employed to monitor the expression levels of pro‐inflammatory cytokines (TNF‐α, IL‐6) and anti‐inflammatory cytokines (IL‐10, Arg‐1) in wound tissues at day 3, 7, and 14. qRT‐PCR data revealed that on day 14 (Figure 7D–G), the hydrogel‐treated group exhibited a significant reduction in IL‐6 and TNF‐α expression and an increase in IL‐10 and Arg‐1 levels relative to the control groups, suggesting an earlier transition from the inflammatory phase to the remodeling phase in wounds treated with the hydrogel.

In the gauze and 3 M groups, the red fluorescence intensity, indicative of pro‐inflammatory cytokine expression levels (IL‐6, TNF‐α), was higher than that of the hydrogel group (Figure S14A–C). However, the green fluorescence intensity, representative of anti‐inflammatory and pro‐repair cytokine expression (IL‐10, Arg‐1), was significantly lower than in the hydrogel group (Figure S14A,D,E). The control group primarily exhibited severe inflammatory responses. The cytokine dysregulation observed in the hydrogel group was ameliorated, characterized primarily by an increase in anti‐inflammatory and pro‐repair cytokines (IL‐10, Arg‐1). Consequently, the hydrogel played a crucial role in promoting wound healing by down‐regulating pro‐inflammatory mediators, up‐regulating anti‐inflammatory mediators, and reducing the duration of the inflammatory phase.

**FIGURE 8:**
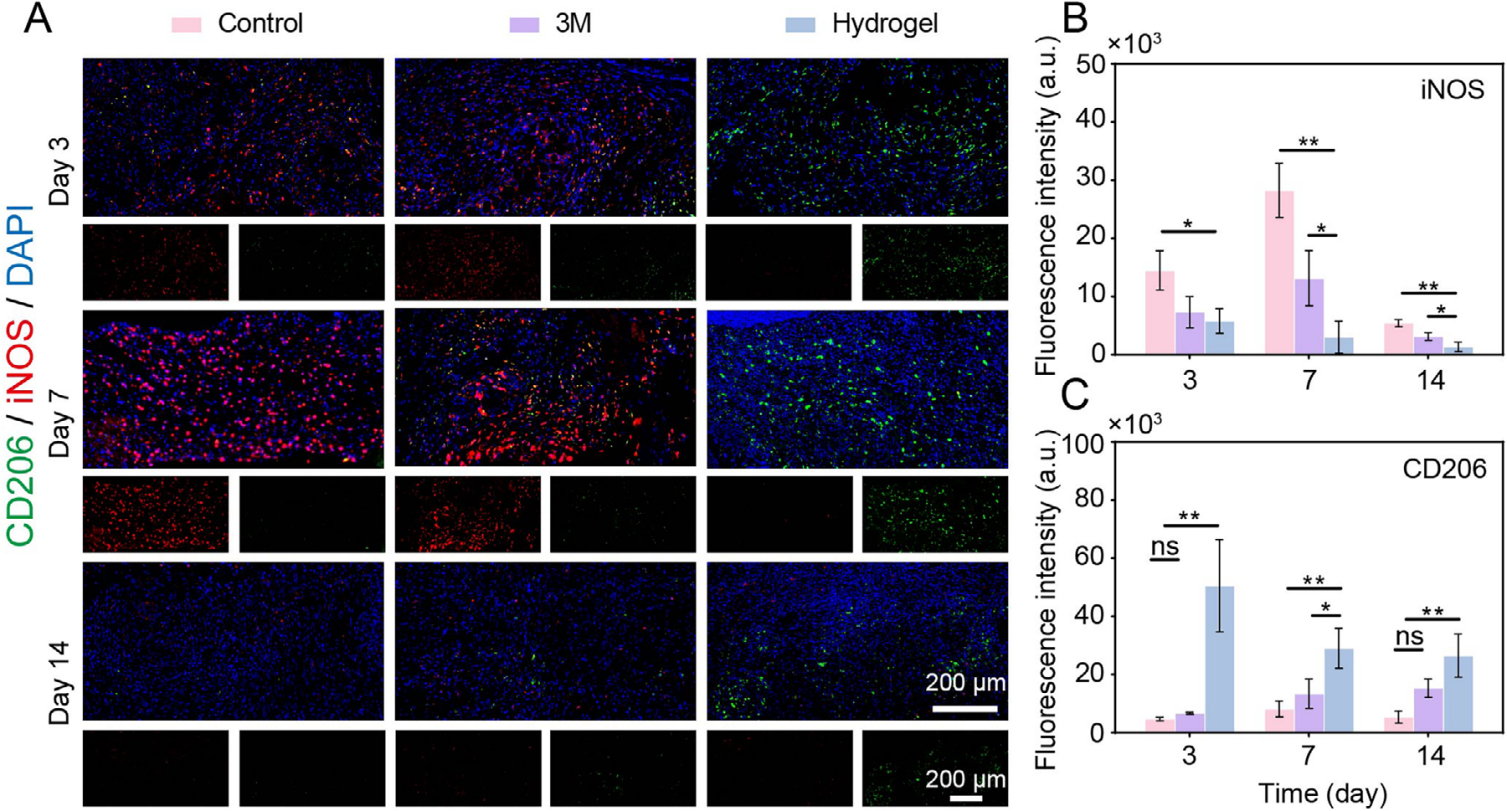
The hydrogel modulates macrophage polarization toward the M2 phenotype in vivo. (A) Representative immunofluorescence images of wound tissues on days 3, 7, and 14, stained for the M1 macrophage marker iNOS (red) and the M2 macrophage marker CD206 (green). Nuclei are counterstained with DAPI (blue). Scale bar: 200 µm. (B, C) Quantitative analysis of the mean fluorescence intensity of iNOS (B) and CD206 (C) across different groups at the indicated time points. Data are presented as mean ± SD (*n* = 3). Statistical significance was assessed using Student's *t*‐tests and one‐way ANOVA; ns, not significant, ^*^ *p* < 0.05, ^**^ *p* < 0.01.

To provide direct cellular phenotypic evidence, we performed immunofluorescence co‐localization staining for iNOS (M1‐specific marker, red) and CD206 (M2‐specific marker, green) on wound tissue sections at postoperative days 3, 7, and 14, with nuclei counterstained using DAPI (blue) (Figure 8A). This enabled direct visualization, localization, and quantification of the spatiotemporal distribution and proportional changes of M1 and M2 phenotype cells. Fluorescence images show that at the early healing stage (day 3), iNOS^+^ M1‐type cells were present in the hydrogel, control, and 3 M groups. However, at critical healing stages (days 7 and 14), the CD206^+^ signal in the hydrogel group was significantly enhanced and enriched, while the iNOS^+^ signal was relatively attenuated. This visually demonstrates the spatial shift of macrophage population from the pro‐inflammatory M1 to the pro‐repair M2 phenotype. We further conducted quantitative analysis of iNOS and CD206 fluorescence intensities (Figure 8B,C). It was observed that CD206 expression (M2 marker) in the hydrogel group was significantly upregulated vs. the control and 3 M groups on days 7 and 14 (*p* <0.01). Simultaneously, iNOS expression (M1 marker) was significantly suppressed at day 14 (p <0.01). The CD206/iNOS ratio increased in the hydrogel group during later stages, further quantifying the overall shift in macrophage polarization. This confirms the dynamic transition of wound macrophages from the M1 to the M2 phenotype.

### 2.6 Histological Analyses of Wound Healing

**FIGURE 9:**
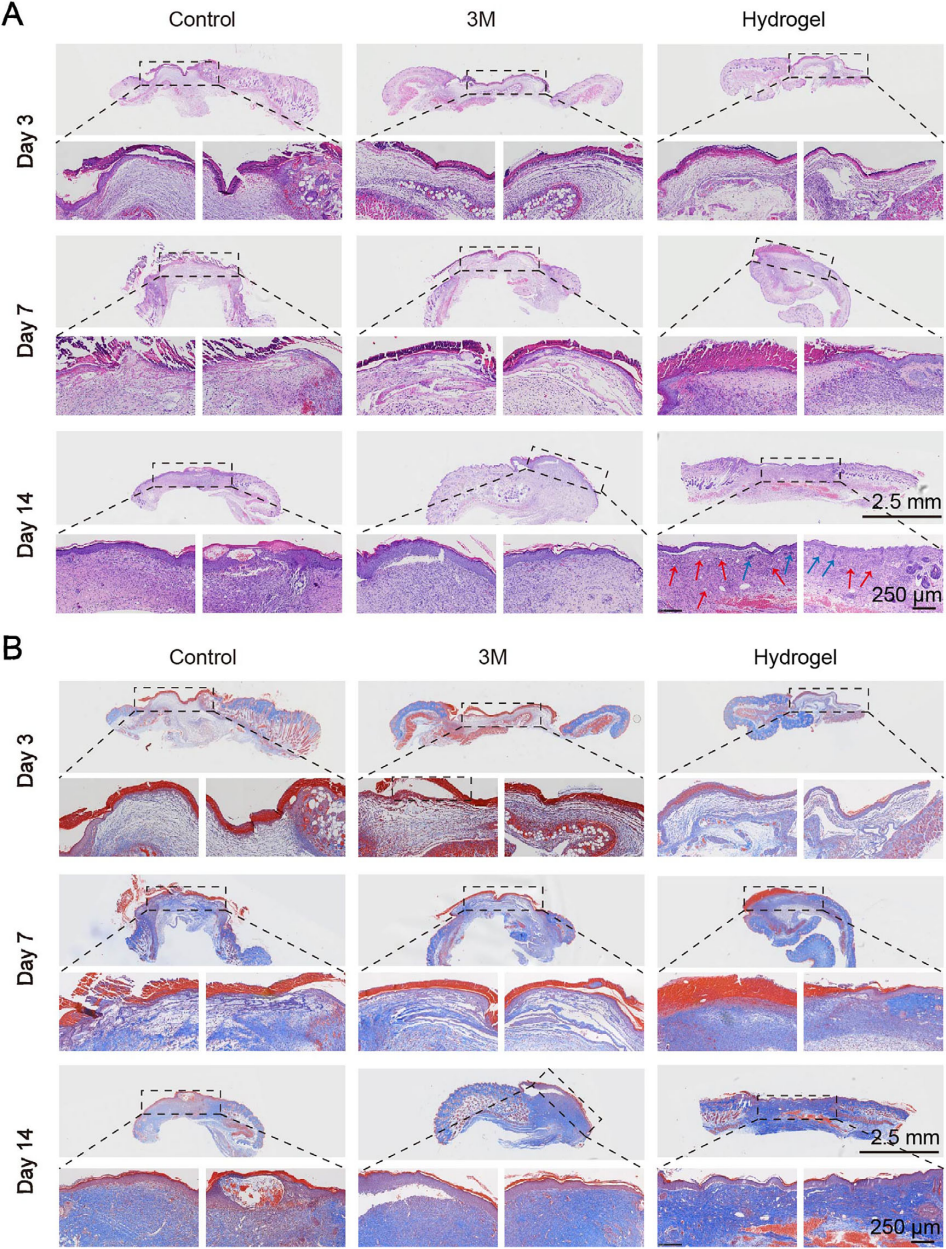
Histological analysis of the wound healing process. (A) H&E staining of wound tissues on days 3, 7, and 14. (B) Masson's trichrome staining of wound tissues on days 3, 7, and 14. Scale bars, as indicated.

H&E staining was used to assess the wound healing process from a histological perspective (Figure 9A). On day 3, pronounced squamous epithelial defects were observed across all groups, accompanied by significant inflammatory exudate and blood scab formation, with the gauze group exhibiting the most pronounced effects. By day 7, the gauze and 3 M groups exhibited larger defects and more severe infiltration of inflammatory cells, whereas the hydrogel group demonstrated good wound healing. Notably, there was a substantial proliferation of new capillaries, granulation tissue formation, and distinct re‐epithelialization. By day 14, complete epithelialization was evident in the hydrogel group. The newly formed epidermis measured 22.04 ± 3.75 µm in thickness (Figure S15), significantly thinner than that of the other control groups (*p* < 0.0001), yet comparable to that of healthy mice (*p* > 0.05), suggesting rapid epidermal maturation. Remarkably, newly formed hair follicles (indicated by blue arrows), newly formed blood vessels (red arrows), and a consistent pattern of collagen deposition were observed beneath the epidermis. Regenerated collagen is crucial to wound repair, as proper collagen deposition enhances tissue tensile strength. It is well established that increased collagen deposition during the proliferative phase and more consistent collagen organization during the remodeling phase are associated with improved wound healing efficiency [77]. Therefore, Masson's trichrome staining was employed to evaluate collagen accumulation and organization during wound healing (Figure 9B). Observations indicated the emergence of new collagen fibers in the hydrogel group as early as the third day, whereas minimal collagen presence was found in other groups. By day 7, collagen fibers in the hydrogel group had significantly increased compared to other groups. On day 14, although all groups showed increased collagen, the hydrogel group exhibited more regular, orderly, complete, and compact fibers, suggesting that the new granulation tissue had transitioned into collagen‐rich subdermal tissue. Consequently, the combined use of Masson's trichrome and H&E staining demonstrated that the hydrogel effectively facilitated wound regeneration and remodeling.

### 2.7 Immunofluorescence Staining and Extracellular Matrix Remodeling Analysis

**FIGURE 10:**
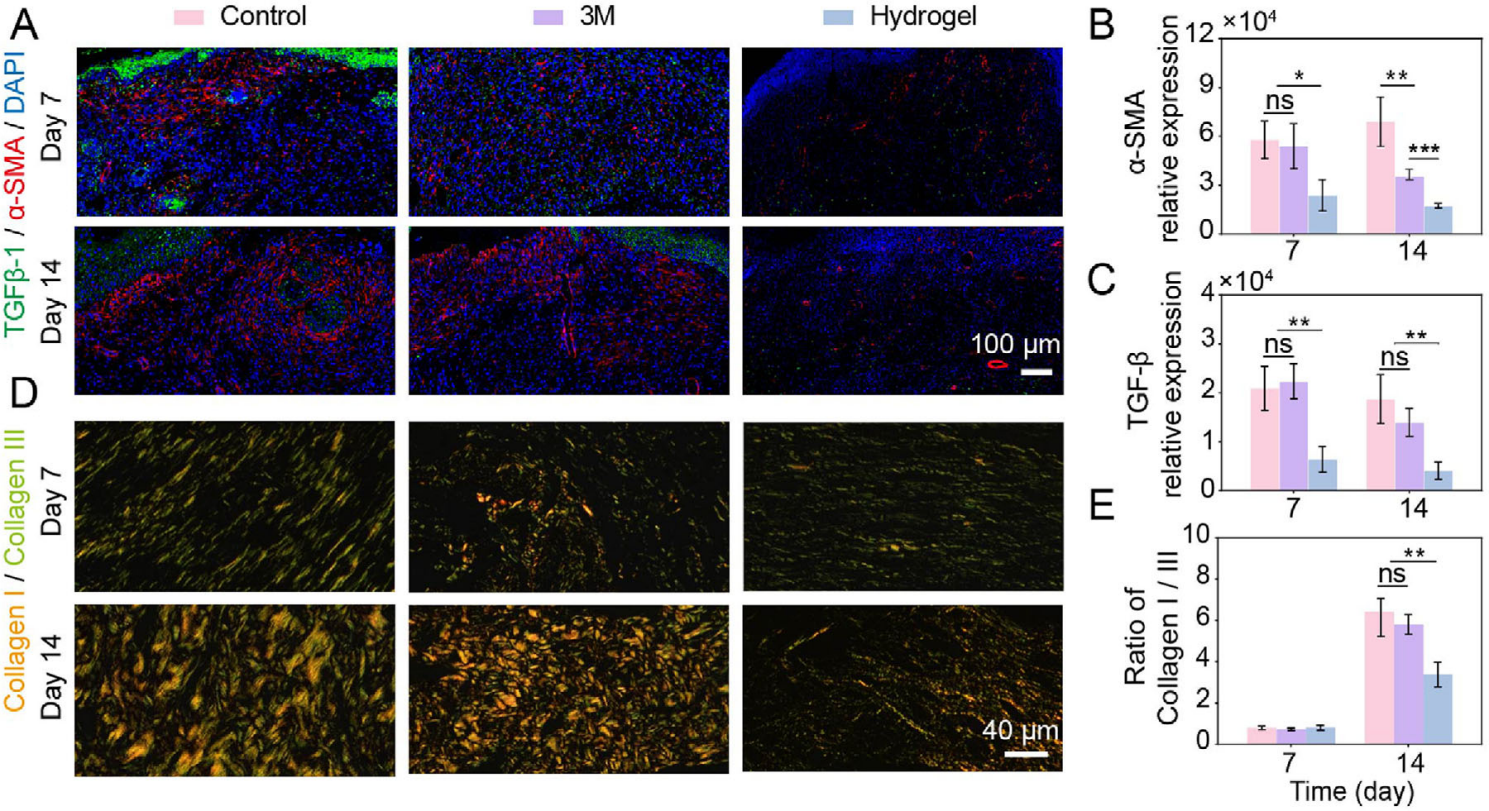
Modulation of fibrotic response and promotion of regenerative extracellular matrix remodeling in vivo. (A–C) Representative immunofluorescence staining and quantitative analysis of α‐SMA and TGF‐β1 in wound tissues on postoperative days 7 and 14. α‐SMA (red) and TGF‐β1 (green) are shown; nuclei are counterstained with DAPI (blue). (D) Representative images of collagen deposition and arrangement. Type I collagen appears orange‐red, and type III collagen appears green. (E) Quantitative analysis of the collagen I/III area ratio. Data are presented as mean ± SD (*n* = 3). Statistical significance was assessed using Student's *t*‐tests and one‐way ANOVA; ns, not significant, ^*^ *p* < 0.05, ^**^ *p* < 0.01, ^***^ *p* < 0.001. Scale bars, as indicated.

To elucidate the cellular and molecular mechanisms by which the hydrogel regulates scar formation, this study employed immunofluorescence double staining to evaluate the expression dynamics of key fibrotic markers and collagen remodeling characteristics (Figure 10). Compared with the control group and the 3 M commercial dressing group, the hydrogel group exhibited significantly reduced fluorescence intensity of α‐SMA (a myofibroblast marker) and TGF‐β1 (a core pro‐fibrotic factor) on postoperative days 7 and 14 (Figure 10A–C). These findings indicate that the hydrogel continuously inhibits the fibrotic process from upstream signaling to downstream effector cell differentiation. Sirius Red staining under polarized light revealed that the hydrogel group had a significantly lower type I/III collagen area ratio than the control group on postoperative day 14 (Figure 10D,E). Additionally, type I collagen deposition was markedly reduced, with fiber arrangement displaying a more organized bundled structure—suggesting that the hydrogel effectively optimizes collagen composition and spatial architecture, thereby promoting extracellular matrix (ECM) remodeling toward a physiological pattern and preventing pathological scar‐like accumulation. These results suggest that the hydrogel regulates the TGF‐β1/α‐SMA signaling axis by modulating macrophage polarization, thereby suppressing abnormal myofibroblast activation and excessive collagen deposition. This guides orderly ECM transformation into a regenerative collagen structure, providing key mechanistic insights into scarless repair. Notably, the observed collagen deposition pattern provides critical evidence for high‐quality regeneration facilitated by the hydrogel. Masson staining revealed that the total collagen area in hydrogel‐treated wounds was significantly higher than that in the control group during the healing, consistent with its function in promoting fibroblast activation and accelerating granulation tissue formation. However, Sirius Red staining under polarized light demonstrated that collagen deposited in the hydrogel group exhibited a significantly lower type I/III ratio, with type I collagen fibers arranged in an orderly manner devoid of nodular aggregation. This phenomenon of “increased quantity with improved quality” indicates that the hydrogel not only promotes the synthesis of all collagen types but also intelligently guides the extracellular matrix toward physiological remodeling resembling that of the normal dermis. Specifically, it provides abundant provisional matrix (predominantly type III collagen) during the repair phase while effectively suppressing pathological over‐deposition and disorganized arrangement of type I collagen during the remodeling phase. This dual regulation of collagen “quantity” and “quality” is central to balance pro‐healing effects with anti‐scarring outcomes.

## 3 Conclusion

The integration of fibrin with dextran‐MA–DMA hydrogel matrices has facilitated the successful synthesis of an interpenetrating hydrogel network. These ingredients impart essential biological functions to the network, notably hemostatic properties and promotion of cellular proliferation. The dextran‐MA component enhances mechanical robustness, allowing for tunable mechanical attributes. The presence of phenolic hydroxyl groups on DMA endows the hydrogel with adhesive properties and the capacity for ROS scavenging. The hydrogel exhibits superior biocompatibility, strong adhesion to wet and slippery wound skin, and effective antioxidant and anti‐inflammatory properties. These characteristics collectively reduce inflammatory responses, enhance cellular proliferation, facilitate collagen deposition, and promote re‐epithelialization, thereby accelerating scarless wound healing. Hence, this work introduces a generalizable design scaffold for bioadhesive hydrogels that couples biological crosslinking with synthetic reinforcement. The enzymatic‐chemical dual‐network strategy can be readily adapted to other protein biomaterial systems, and the tunable mechanical and adhesive properties make it suitable for diverse irregular internal wound scenarios, including soft‐tissue repair, internal bleeding control, and postoperative sealing.

## 4 Experimental Section

### 4.1 Synthesis of Dextran‐MA

Glycidyl methacrylate (GMA) was first distilled to remove the hydroquinone stabilizer. Under an inert atmosphere, dextran (10.02 g) was dissolved in anhydrous dimethyl sulfoxide (70 mL). 4‐Dimethylaminopyridine (2.00 g) and distilled GMA (10 mL) were added, and the mixture was stirred at room temperature for 48 h. After quenching, the solution was neutralized to pH 7 and dialyzed against deionized water at 4°C for 5 days. The product was isolated as a white powder by freeze‐drying (Figure S1) [33].

### 4.2 Preparation of Fibrin–Dextran‐MA–DMA Hydrogel

Fibrinogen, dextran‐MA, CaCl_2_, DTT, thrombin, and DMA were dissolved in distilled water at the ratios given in Table S1. The mixture was degassed under vacuum and then incubated at 37°C for 1 h to allow complete gelation.

### 4.3 Adhesion Test

Hydrogel adhesion was measured using a universal testing machine (EZ‐LX, Shimadzu, Japan) in lap‐shear mode. Glass or pigskin substrates were overlapped to create a butt‐shear joint of a fixed area and tested at a speed of 5 mm/min until failure. Adhesive strength (τ) was calculated as follows:

$$\tau =\frac{\mathrm{Maximum}\;\mathrm{load}\;\left(F\right)}{\mathrm{Bonding}\;\mathrm{area}\;\left(A\right)}$$

Three measurements were performed for each sample and averaged.

### 4.4 Rheological Measurements

Rheological characterization was performed on a HAAKE RS6000 rheometer (Germany) using a 35‐mm parallel‐plate geometry at 37°C. Three scan types were run sequentially: time sweep, frequency sweep, and strain sweep. In the time‐sweep experiment, the frequency was fixed at 1 Hz, and the shear stress at 1 Pa. Oscillatory frequency sweeps (shear stress = 1 Pa, 0.1–100 Hz) yielded the storage modulus (*G*′) and loss modulus (*G*″) as functions of frequency. Oscillatory strain sweeps (frequency = 1 Hz, 0.1%–1000% strain) defined the linear viscoelastic region and the corresponding moduli [36].

### 4.5 Swelling Characterization

Shape‐identical hydrogels were immersed in PBS for 24 h and then weighed (*w*). After freeze‐drying, the dry weight (*w*_0_) was recorded. The swelling ratio (*Q*) of the hydrogels was calculated using the following formula:

$$Q=\frac{w-w_{0}}{w_{0}}\;\times 100$$

### 4.6 Surface Morphology Characterization

Surface morphologies of the hydrogels were characterized using a scanning electron microscope (SEM, JEOL, Japan) (Figure S4).

### 4.7 Preparation of Hydrogel Extract

Freshly prepared hydrogel (0.2 g/mL) was immersed in DMEM for 24 h, and the resulting extract was supplemented with 10% FBS. The medium was then sterilized by filtration through a 0.22 µm membrane, yielding the sterile hydrogel extract.

### 4.8 In Vitro Cytocompatibility

L929 mouse fibroblasts were seeded into 48‐well plates (8 × 10^3^ cells per well, 200 µL, *n* = 3) containing the extract and incubated at 37°C, 5% CO_2_. After 24, 48, and 72 h, the medium was replaced with 200 µL fresh DMEM plus 20 µL CCK‐8 reagent; absorbance at 450 nm was read (Tecan, Switzerland) after 1 h. Parallel wells were stained with the LIVE/DEAD Viability/Cytotoxicity Kit and imaged on a Nikon ECLIPSE Ti2‐E inverted microscope (Nikon, Japan) at different time points.

### 4.9 Determination of RGD Concentration in Hydrogel Extract by ELISA

The sterile hydrogel extract, prepared as described in the “Preparation of Hydrogel Extract” section, was centrifuged at 4°C and 12 000 rpm for 10 min, and the supernatant was collected as the test sample. The Human RGD Tripeptide ELISA Kit (Shanghai Sentai Biotechnology Co., Ltd.) was employed for detection. Measurements were performed on a multi‐mode microplate reader (Tecan, Switzerland) with the wavelength set to 450 nm. A standard curve was plotted with the standard concentration as the abscissa and the OD value as the ordinate using four‑parameter logistic (4‐PL) curve fitting (R^2^ >0.99). The RGD concentrations of the samples were calculated from their OD values using the standard curve. The final results are expressed as mean ± standard deviation (*n* = 6) (Figure S5A).

### 4.10 In Vivo Biosafety Evaluation

During the hydrogel degradation study, male BALB/c mice received a subcutaneous injection of the hydrogel into the dorsal skin. Heart, liver, spleen, lung, and kidney were harvested on days 3, 7, 14, 30, and 50, processed for H&E staining, and examined under an optical microscope (Olympus, Japan) to assess systemic toxicity. All animal experiments were reviewed and approved by the Ethics Committee of LZU No. 1 Hospital prior to the research (approval number: LDYYLL‐2025‐2019).

### 4.11 In Vitro Hemolysis Rate

The test was performed in accordance with ISO 10993–4:2017 and ASTM F756‐17. Fresh Sprague‐Dawley rat (SD rat) whole blood was diluted 1: 1.25 (*v*/*v*) with sterile 0.9% saline and gently mixed. Negative control (0.9% saline), positive control (distilled water), and hydrogel extract were pre‐warmed to 37°C for 30 min. Subsequently, 0.1 mL of the diluted blood was added to 0.9 mL of each pre‐warmed sample (n ≥ 3) and incubated at 37°C for 60 min. After centrifugation at 2500 rpm for 5 min, 200 µL of the supernatant was transferred to a 96‐well plate, and the absorbance at 545 nm was measured using a microplate reader (Tecan, Switzerland). The hemolysis rate was calculated as:

$$\mathrm{Hemolysis}\;\left(\%\right)=\frac{OD_{\mathrm{hydrogel}}-OD_{\mathrm{negative}\;\mathrm{control}}}{OD_{\mathrm{positive}\;\mathrm{control}}-OD_{\mathrm{negative}\;\mathrm{control}}}\times 100\%$$

### 4.12 Cell Migration Assay

A marker pen was used to draw horizontal lines on the underside of each well in a 6‐well plate, ensuring that at least 5 lines crossed each well. Approximately 1 × 10^5^ cells were seeded per well and incubated overnight. A straight scratch was then made perpendicular to the lines with a sterile 200 µL pipette tip. After scratching, the wells were gently washed with PBS to remove detached cells and refilled with serum‐free medium. Cells in the experimental group were cultured using the serum‐free hydrogel extract, while cells in the control group were treated with serum‐free medium. The plates were incubated at 37°C, 5% CO_2_. Migration was assessed at 24 and 48 h with a Nikon ECLIPSE Ti2‐E inverted microscope (Nikon, Japan). Images were captured, and the scratch width was measured at ≥3 sites per well using ImageJ.

### 4.13 In Vitro Degradation Study

Circular hydrogels of identical size were lyophilized and weighed (*w*₀), then immersed in 1 mg/mL dextranase solution at 37°C. At predetermined intervals, samples were rinsed with deionized water, lyophilized, and re‐weighed (*w*_t_). This procedure was repeated at each time point. The remaining mass was calculated as:

$$\mathrm{Remaining}\;\mathrm{Mass}\;\left(\%\right)=\frac{1-\left(w_{0}-w_{t}\right)}{w_{0}}\times 100\%$$

### 4.14 In Vivo Degradation Assay

Before gelation, 500 µL of pre‐gel solution was injected subcutaneously into the dorsal skin of male BALB/c mice using a sterile 1 mL syringe. At days 3, 7, 14, 30, and 50, the remaining hydrogel was explanted, photographed, and weighed (*n* = 3). Residual mass was calculated as:

$$\mathrm{Remaining}\;\mathrm{Mass}\;\left(\%\right)=\frac{\mathrm{Current}\;\mathrm{Weight}}{\;\mathrm{Initial}\;\mathrm{Weight}}\times 100\%$$

### 4.15 Fourier Transform Infrared (FTIR)

Fourier transform infrared (FTIR) spectroscopy was employed to characterize the chemical structure of the hydrogel degradation products. The freeze‐dried degradation products were ground and mixed with potassium bromide (KBr), then pressed into pellets. FTIR spectra were recorded over a wavenumber range of 4000–500 cm^−^^1^ with 32 cumulative scans. A background spectrum was collected prior to each measurement to eliminate environmental interference.

### 4.16 Nuclear Magnetic Resonance (NMR)

The chemical structure and composition of the degradation products were further analyzed by nuclear magnetic resonance (NMR) spectroscopy. The freeze‐dried degradation products were dissolved in an appropriate amount of deuterium oxide (D_2_O) and transferred into an NMR tube for analysis. ^1^H NMR spectra were recorded at room temperature using an NMR spectrometer. Chemical shifts were calibrated using the residual solvent peak as an internal standard and are expressed in parts per million (ppm).

### 4.17 In Vitro Pro‐Coagulation Performance

The hemostatic capability of the fibrin–dextran‐MA–DMA hydrogel was evaluated by determining the whole‐blood clotting index (BCI) and clotting time, with sterile medical gauze and a commercial gelatin hemostatic sponge (Jiangxi Xiang'en Medical Technology Development Co., Ltd., China) as controls.

### 4.18 BCI Measurement

Fresh anticoagulated whole blood (sodium citrate) was recalcified with 0.1 M CaCl_2_ prior to testing. Then, 60 µL of the recalcified whole blood was placed onto the surface of the test material (or blank control) and incubated at 37°C for 10 min. After incubation, 5 mL of deionized water was added to lyse unincorporated red blood cells. The resulting liquid was collected, centrifuged, and the absorbance at 545 nm was recorded (*OD*_sample_). Whole blood without any material (*OD*_blood_) and supernatant from completely clotted blood (*OD*_supernatant_) served as the 0% and 100% coagulation references, respectively. BCI was calculated as:

$$\mathrm{BCI}\;\left(\%\right)=\frac{OD_{sa\mathrm{mple}}-OD_{\mathrm{supernatant}}}{OD_{\mathrm{blood}}-OD_{\mathrm{supernatant}}}\times 100\%$$

### 4.19 In Vivo Hemostasis Properties

The in vivo hemostatic performance of the fibrin–dextran‐MA–DMA hydrogel was evaluated in SD rats using liver injury and tail amputation models, with sterile medical gauze and a commercial gelatin hemostatic sponge as controls. SPF‐grade male SD rats (250–300 g) were anesthetized and placed in the supine position. After abdominal disinfection, a 3‐cm midline incision was made to expose the liver. A liver lobe was dried with sterile gauze and positioned on pre‐weighed filter paper. A 10‐mm‐long, 2‐mm‐deep incision was made in the liver surface with a scalpel. After 3 s of free bleeding, the pre‐prepared hydrogel was applied to the wound. Bleeding time was recorded with a stopwatch, and blood loss was quantified by the weight gain of the filter paper (*n* = 3). For the tail‐amputation model, the distal third of the tail was removed under anesthesia. After 3 s of bleeding, the tail was immediately placed into a pre‐weighed glass bottle containing the testing material. Complete hemostasis time was recorded, and blood loss was determined gravimetrically (*n* = 3).

### 4.20 Antioxidant Activity

Hydrogels (3 mg) were incubated with 3 mL of 1 mM 1,1‐diphenyl‐2‐picrylhydrazyl (DPPH) in ethanol for 3 h at room temperature in the dark. The optical OD of the supernatant was then measured at 517 nm. The calculation formula was:

$$D=\left(1-\frac{As-Ao}{Ac}\right)\times 100\%$$

where *A*_o_ is the optical density of the hydrogel in ethanol without DPPH, *A*c is the optical density of the DPPH solution alone, and *A*_s_ is the optical density of the DPPH solution after sample treatment.

For the 2‐phenyl‐4,4,5,5‐tetramethylimidazoline‐3‐oxide‐1‐oxyl (PTIO) assay, 3 mg of PTIO powder was dissolved in 20 mL distilled water to prepare the test solution, subsequent steps were identical to those of the DPPH assay.

### 4.21 Cellular ROS‐Scavenging Assay

The hydrogel's intracellular ROS‐scavenging capacity was assessed with the DCFH‐DA probe. RAW264.7 cells were seeded in 6‐well plates and cultured for 24 h. The experimental group received hydrogel extract supplemented with 100 µM Rosup, the control group received 100 µM Rosup alone, and an untreated group served as the negative control. After 1 h, cells were washed with PBS, incubated with DCFH‐DA working solution for 30 min, and imaged by fluorescence microscopy. Mean fluorescence intensity was quantified with ImageJ.

### 4.22 In Vitro Anti‐Inflammatory Study

A 0.1 µg/mL LPS working solution was prepared by dissolving 1 mg LPS powder in DMEM + 10% FBS to 0.1 mg/mL, sterile‐filtering, and then diluting 10 µL of this stock into 10 mL DMEM + 10% FBS. RAW 264.7 cells (1 × 10^6^) were suspended in 2 mL hydrogel extract containing 0.1 µg/mL LPS, seeded into 6‐well plates, incubated, and stimulated for 48 h. Untreated cells served as the blank, and cells exposed to 0.1 µg/mL LPS alone as the positive control. After treatment, cells were washed with 1 mL PBS, pelleted by centrifugation (12 000 × g, 4°C, 5 min), and the supernatant was discarded. Total RNA was extracted with 1 mL Trizol reagent and reverse‐transcribed into cDNA using the PrimeScript RT reagent kit under the following conditions: 42°C for 15 min, 85°C for 5 s, and cDNA was stored at −20°C. Gene expression was quantified by qRT‐PCR in a 20 µL reaction containing 10 µL TB Green Premix Ex Taq II, 0.4 µL ROX reference dye, 6 µL RNase‐free water, 2 µL cDNA, and 1.6 µL primers (0.8 µL each of forward and reverse primer, Table S2). The qRT‐PCR program consisted of an initial denaturation at 95°C for 30 s, followed by 40 cycles of 95°C for 5 s and 60°C for 34 s. A dissociation curve was generated by heating to 95°C for 15 s, cooling to 60°C for 1 min, and reheating to 95°C for 15 s. The expression levels of pro‐inflammatory factors IL‐6 and TNF‐α, and anti‐inflammatory factors IL‐10 and Arg‐1, were calculated using cycle threshold (Ct) values relative to the endogenous housekeeping gene (β‐actin).

### 4.23 RNA Sequencing (RNA‐seq) and Data Analysis

Total RNA was extracted as described above. Sequencing libraries were constructed using the VAHTSTM mRNA‐seq V2 Library Prep Kit for Illumina, and library quality was evaluated on the Agilent Bioanalyzer 2100 system. Raw reads were first quality‐checked using FastQC, then aligned to the reference genome with HISAT2; mapping statistics were computed with samtools. Aligned reads from all samples were jointly assembled by StringTie, and the resulting transcripts were compared against annotated gene models with gffcompare to identify novel loci. Gene‐level counts were obtained with featureCounts using the reference annotation. The count matrix was used for exploratory analyses and differential expression (DE) testing with DESeq2. DE results were visualized with ggplot2. Gene Ontology (GO) enrichment was performed using topGO and KEGG pathway enrichment with clusterProfiler.

### 4.24 Wound Healing

The healing effects of the hydrogel on wounds were evaluated using a full‐thickness skin wound model in mice. Male BALB/c mice (18–22 g) were randomly assigned to three groups (*n* = 5): the gauze group, the medical 3 M wound dressing group, and the hydrogel group. After anesthesia and dorsal shaving, an 8‐mm circular full‐thickness wound was created on each mouse. Wounds were covered as follows:

Gauze group: gauze + 3 M Tegaderm film + adhesive tape.

3 M group: medical 3 M wound dressings + 3 M Tegaderm film + adhesive tape.

Hydrogel group: Fibrin–dextran‐MA–DMA hydrogel + 3 M Tegaderm film + adhesive tape.

Wounds were photographed on days 0, 3, 7, and 14. Wound areas were measured with ImageJ and the healing rate was calculated as:

$$\begin{array}{cc} & \mathrm{Wound}\;\mathrm{healing}\;\mathrm{rat}\\ & =\frac{\mathrm{wound}\;\mathrm{area}\;\mathrm{on}\;\mathrm{day}\;0-\mathrm{wound}\;\mathrm{area}\;\mathrm{on}\;\mathrm{certain}\;\mathrm{day}}{\mathrm{wound}\;\mathrm{area}\;\mathrm{on}\;\mathrm{day}\;0}\times 100\% \end{array}$$

Samples from each skin tissue were collected on days 3, 7, and 14, and divided into two groups. One group was fixed with 4% formaldehyde, dehydrated, and then embedded in paraffin. The sections were cut to a thickness of 4 µm and used for HE staining, Masson trichrome staining, Sirius Red Staining, and immunofluorescence staining of IL‐6, TNF‐α, IL‐10, Arg‐1, iNOS, and CD206. Both HE and Masson slices were imaged by a WISLEAP WS‐10 Pathology Slide Scanner, and the immunofluorescence slices were imaged in a NIKON ECLIPSE Ti2‐E inverted microscope (Nikon, Japan). The other group of tissues was immersed in Trizol reagent, and the expression levels of IL‐6, TNF‐α, IL‐10, and Arg‐1 were detected with qRT‐PCR.

### 4.25 qRT‐PCR

On days 3, 7, and 14 of the mouse wound experiment, skin wound tissues were collected. The mRNA expression levels of IL‐6, TNF‐α, IL‐10, and Arg‐1 were measured by qRT‐PCR. Tissues were immersed in Trizol reagent, minced with ophthalmic scissors, and homogenized with an ultrasonic tissue homogenizer. RNA extraction and qRT‐PCR procedures were identical to those described for the cell experiments.

### 4.26 Statistical Analysis

All in vitro experiments were performed in triplicate. Data are presented as mean ± SD. Data preprocessing and sample size (n) for each analysis were stated in the relevant figure legends. Statistical significance was assessed using Student's *t*‐tests and one‐way ANOVA. *p* < 0.05 was considered statistically significant. ns, not significant. ^*^ *p* < 0.05, ^**^ *p* < 0.01, ^***^ *p* < 0.001, ^****^ *p* < 0.0001.

## Author Contributions

Conceptualization: B. Li. Methodology: X. Li, G. Zhang, X. Wang, Y. Bao. Investigation: X. Li, G. Zhang, M. Wei, L. Yang, Z. Liu, Y. Ma. Visualization: Y. Ma, W. Sheng, B. Yu. Supervision: Y. Chen, B. Li. Writing – original draft: X. Li, B. Li. Writing – review & editing: Y. Chen, B. Li.

## Ethics Statement

All animal experiments were reviewed and approved by the Ethics Committee of LZU No. 1 Hospital prior to the research (approval number: LDYYLL‐2025‐2019).

## Conflicts of Interest

The authors declare no conflicts of interest.

## Supporting information




**Supporting File**: advs75324‐sup‐0001‐SuppMat.docx.

## Data Availability

The data that supports the findings of this study are available in the supplementary material of this article.
