# Supplementary material for: Dual‐Network Protein Hydrogels Promote Rapid Hemostasis and Immune‐Regulated Scarless Tissue Regeneration
Source: Adv Sci (Weinh). 2026 Apr 20;13(38):e75324. doi: 10.1002/advs.75324 (PMC13335640; doi:10.1002/advs.75324)
Supplement: Supplementary file 1 — Supporting File: advs75324‐sup‐0001‐SuppMat.docx. [file ADVS-13-e75324-s001.docx]

Supporting Information

Dual-Network Protein Hydrogels Promote Rapid Hemostasis and Immune-Regulated Scarless Tissue Regeneration

*Xiaomei Li^1,2^, Guorui Zhang^2^, Xinyue Wang^2^, Zhongyu Liu^2^, Ling Yang^2^, Yuhan Bao^2^, Min Wei^2^, Yonglin Chen^1*^, Yanfei Ma^2,3*^, Wenbo Sheng^2,3^, Bo Yu^2^, Bin Li^2,3*^*

^1^The First Clinical Medical College of Lanzhou University, Lanzhou 730000, China

^2^State Key Laboratory of Solid Lubrication, Lanzhou Institute of Chemical Physics, Chinese Academy of Sciences. Lanzhou 730000, China.

^3^Shandong Laboratory of Advanced Materials and Green Manufacturing at Yantai, Shandong 264006, China

*Email: ldyy_chenyl@lzu.edu.cn; mayanfei@licp.cas.cn; binli@licp.cas.cn

Materials

Dextran 150 kDa (VWR International), glycidyl methacrylate (Sigma Aldrich). 4-dimethylaminopyridine (Sigma Aldrich), dimethyl sulfoxide (Sigma Aldrich), fibrinogen (35-65% protein Sigma Aldrich), and thrombin (≥1000 NIH units mg-1 protein Sigma Aldrich). DTT (Carbolution), phosphate buffer saline (PBS, Servicebio), 2-(N-Morpholino) ethanesulfonic acid (MES, J&K), DMEM (Servicebio), Fetal Bovine Serum (FBS, Servicebio), Cell Counting Kit-8 (CCK-8, Servicebio), LIVE&DEAD Viability/Cytotoxicity Assay Kit (Servicebio), Lipopolysaccharide (LPS, Servicebio), Trizol reagent (Thermo Fisher Scientific), PrimeScript^TM^ RT reagent Kit (Takara), TB Green Premix Ex Taq^TM^ II (Takara), 3M Tegaderm^TM^ Film, IL-6 Polyclonal Antibody (Servicebio), IL-10 Polyclonal Antibody (Servicebio), ARG-1 Polyclonal Antibody (Servicebio), TNF-α Polyclonal Antibody (Immunoway).

Supporting Figures


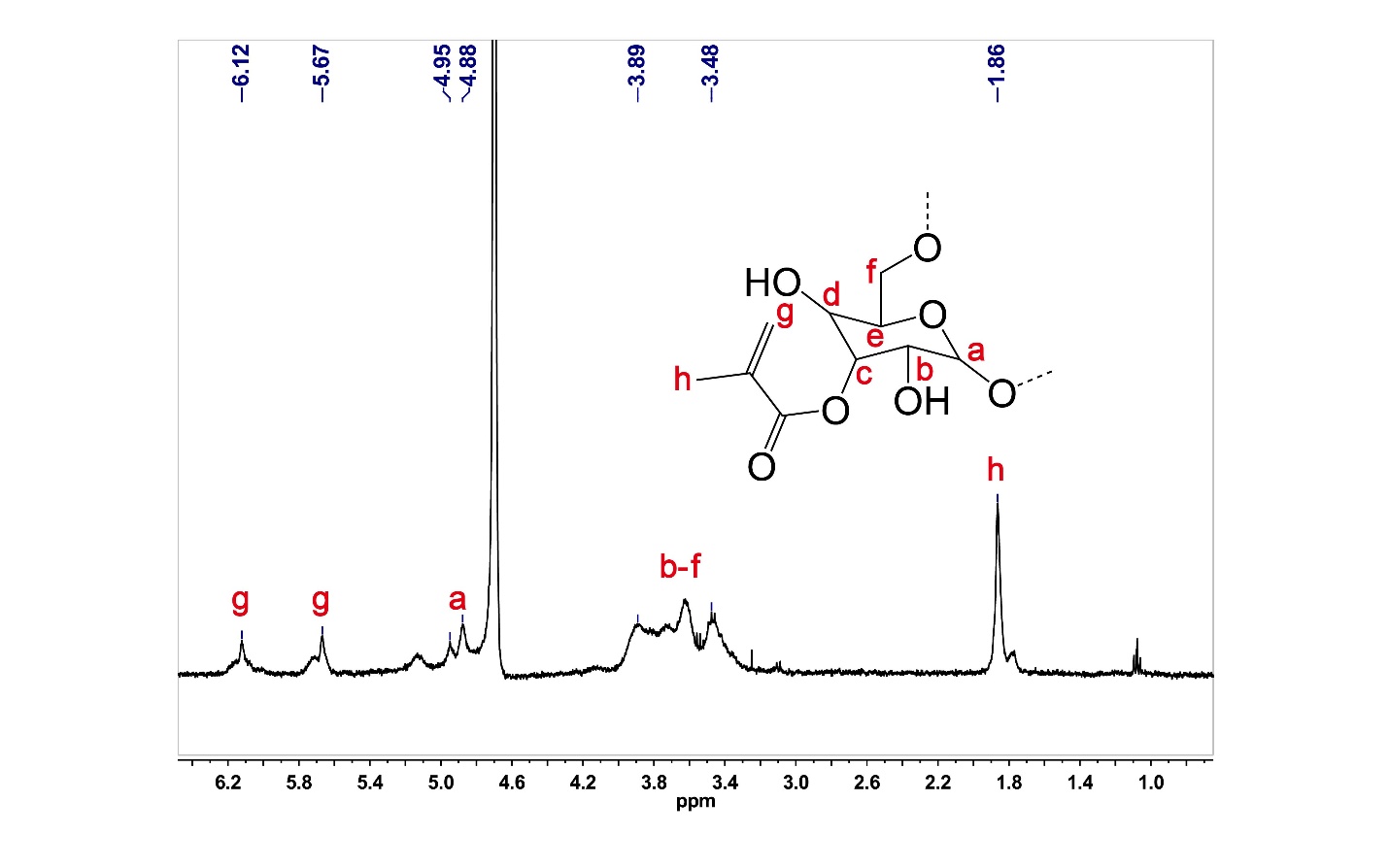
 **FIGURE S1.** ^1^H-NMR of dextran-MA.


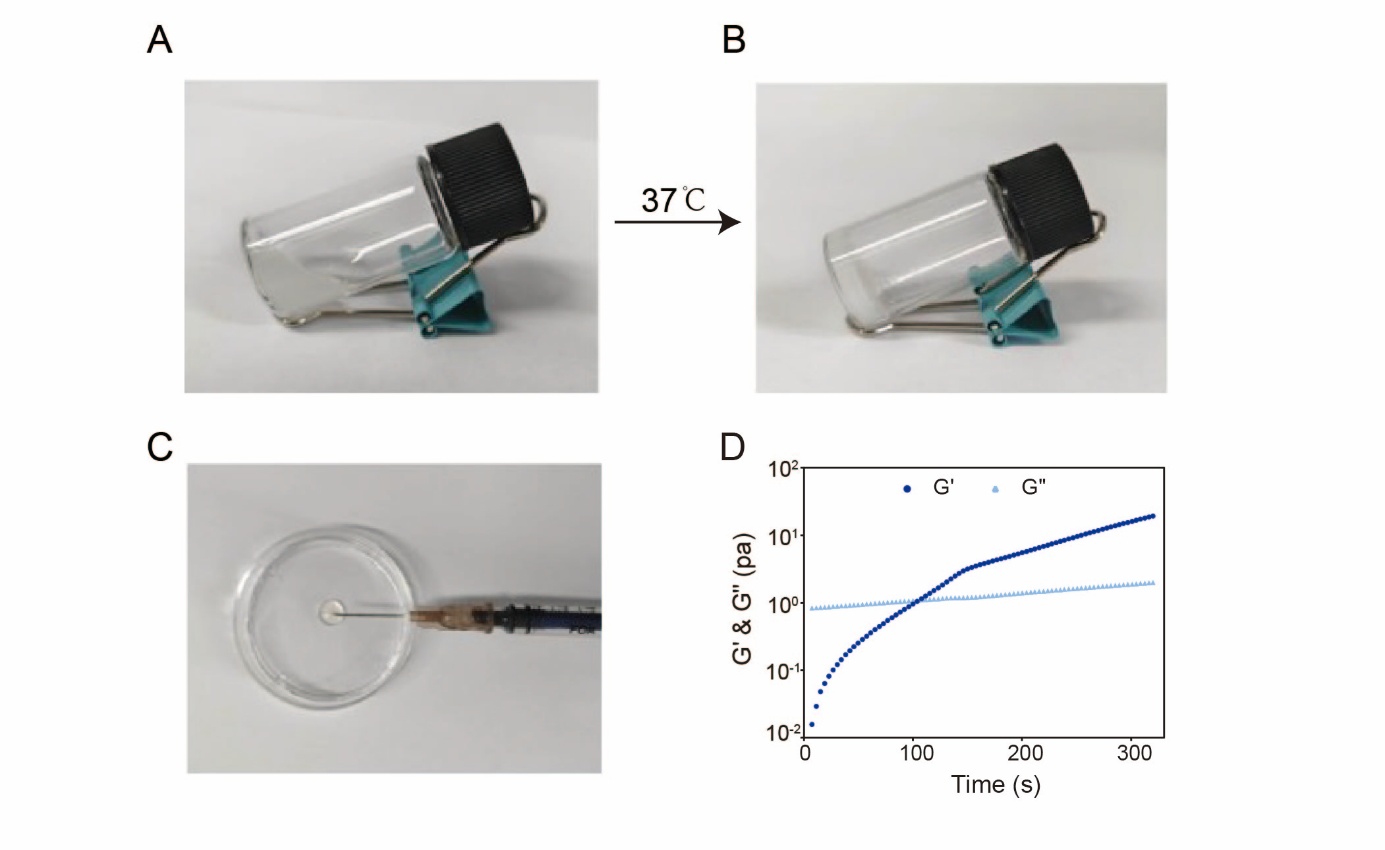


**FIGURE S2.** Photograph of the (A) hydrogel precursor solution, (B) formed hydrogel, (C) injectability of hydrogels, (D) Rheological time‑sweep measurement at 37 °C, showing the evolution of storage modulus (*G*′, solid symbols) and loss modulus (*G*″, open symbols) over time, confirming rapid hydrogel formation under physiological temperature.


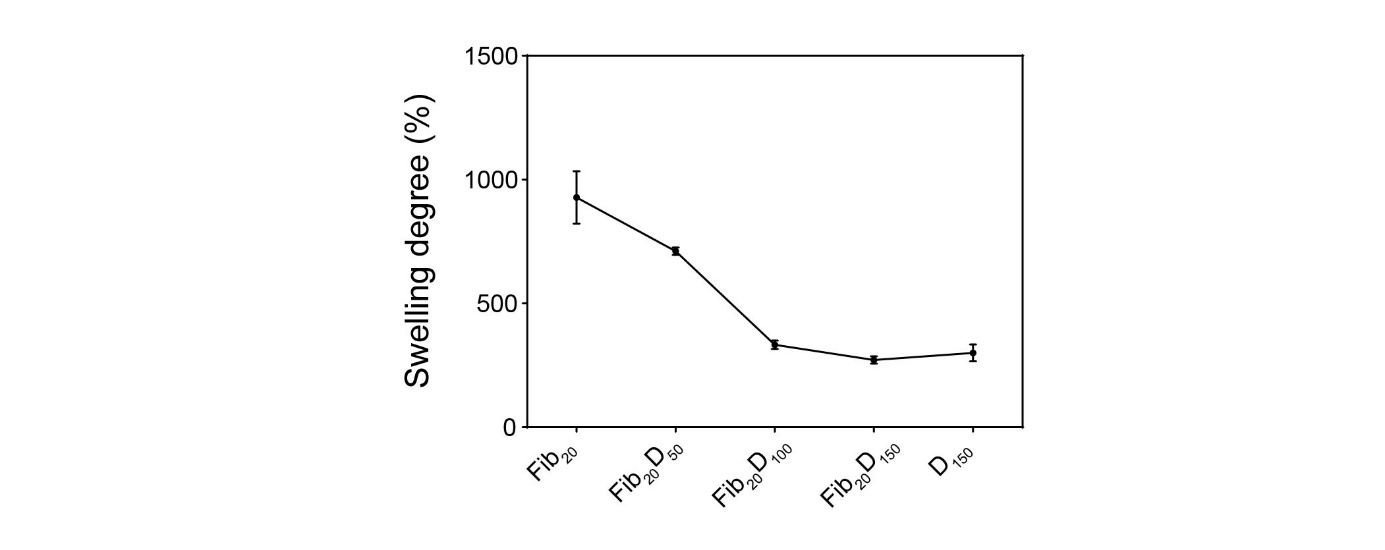


**FIGURE S3.** Swelling degree of hydrogels with different components of fibrin and dextran-MA, n = 3. Data are presented as mean ± SD (n = 3).

**
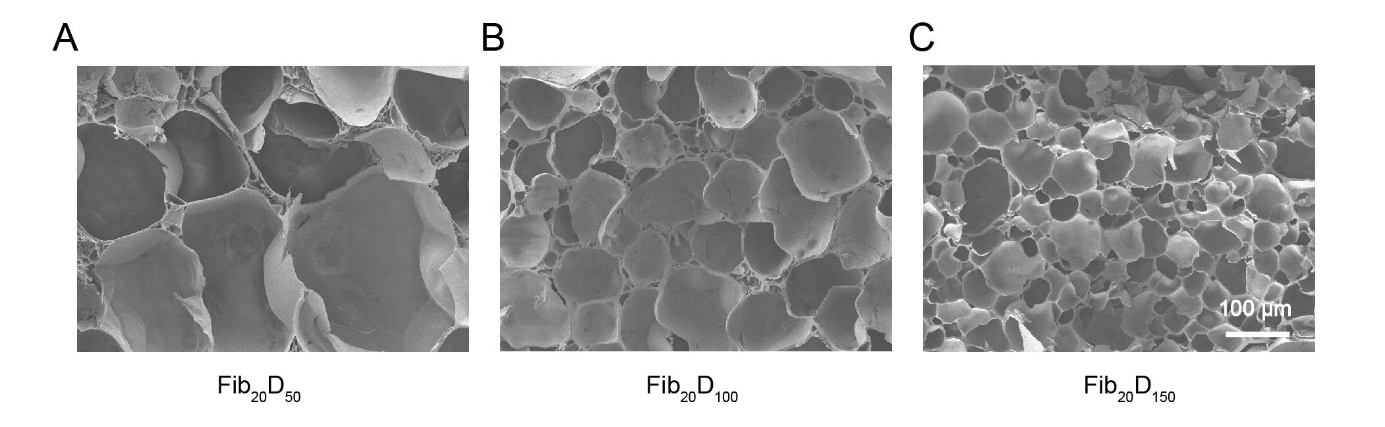
**

**FIGURE S4.** Cross-sectional SEM images of hydrogels with varying fibrin and dextran-MA compositions. F_20_D_50_: dextran-MA (50 mg/mL); F_20_D_100_: dextran-MA (100 mg/mL); F_20_D_150_: dextran-MA (150 mg/mL); fibrinogen (20 mg/mL), thrombin (20 U/mL), DTT (5 mg/mL), DMA (1.86 mg/mL). Scale bar: 100 μm.


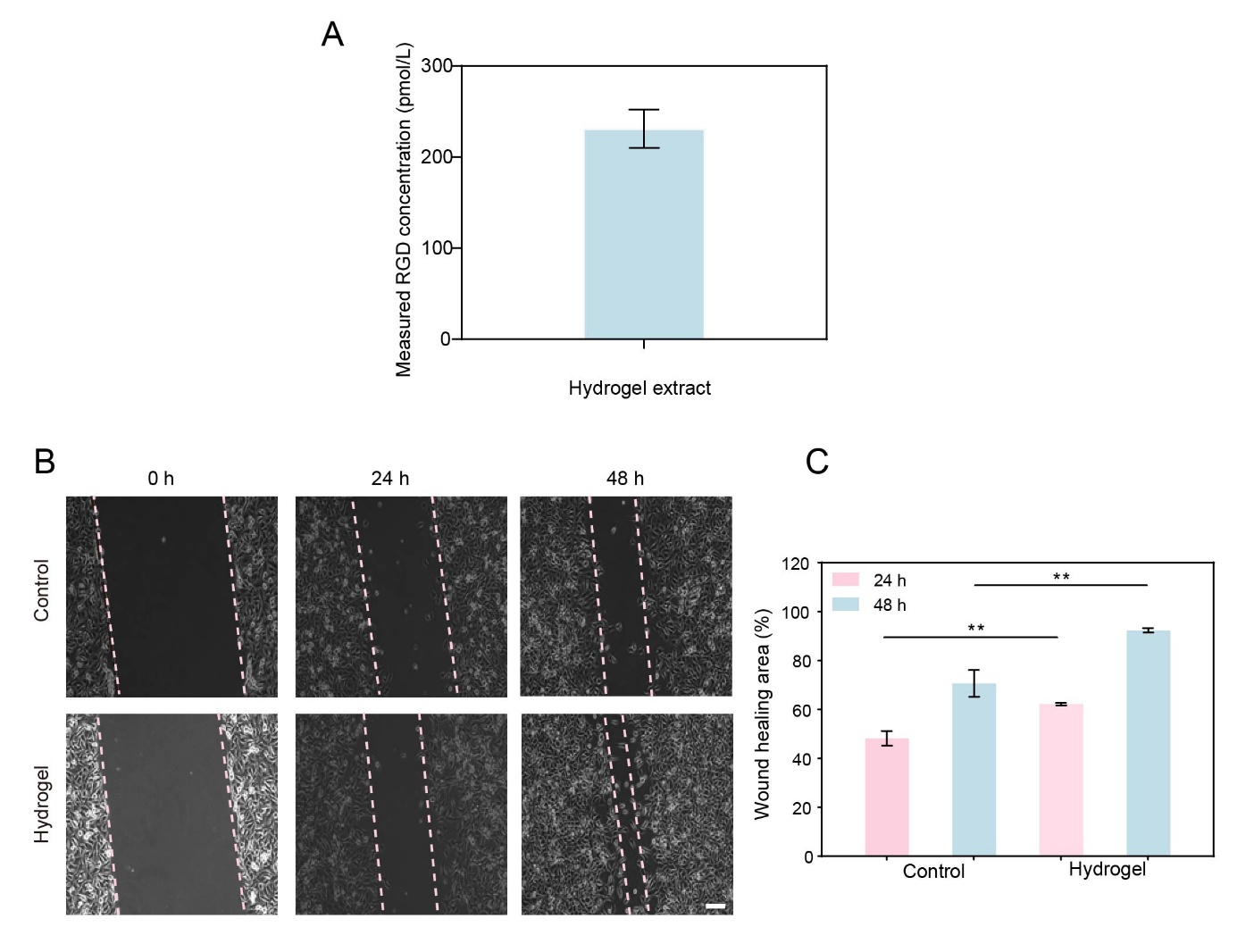


**FIGURE S5.** (A) Concentration of soluble RGD peptides in the hydrogel extract (n = 6). (B) Cell migration experiment of MUVEC cells for 0h, 24 h, and 48 h. Scale bar: 100 μm. (C) Cell migration rate statistics (n = 3). Data are presented as mean ± SD. Statistical significance was assessed using Student’s t-tests; **P <0.01.


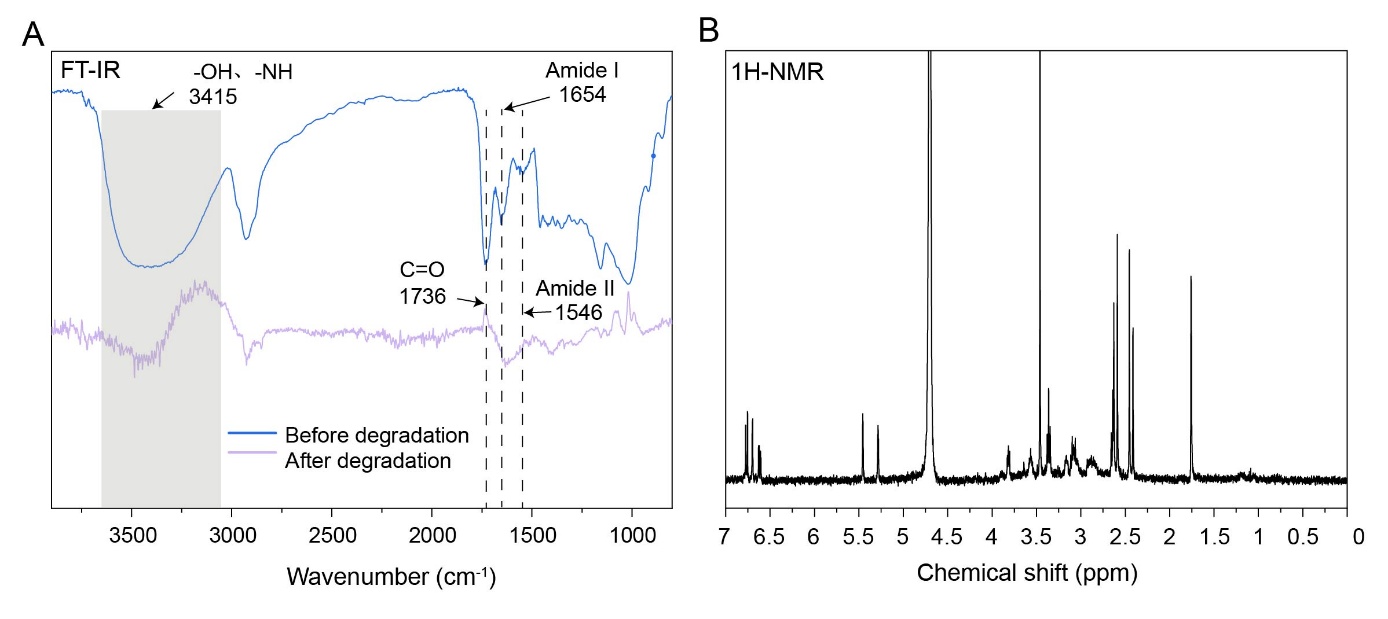


**FIGURE S6.** FT-IR and ¹H NMR characterization of the hydrogel degradation products. (A) FT-IR spectra of the hydrogel before and after degradation. (B) ¹H NMR spectrum of the hydrogel after degradation.


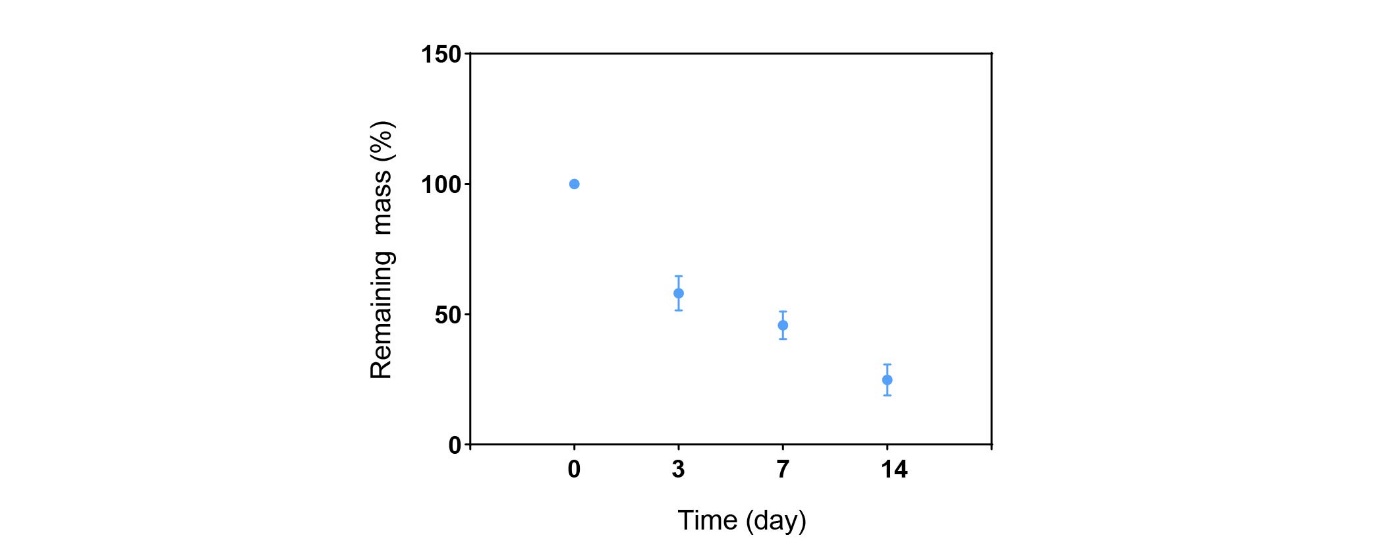


**FIGURE S7.** Mass loss of hydrogels during wound degradation. Data are presented as mean ± SD (n = 3).

**
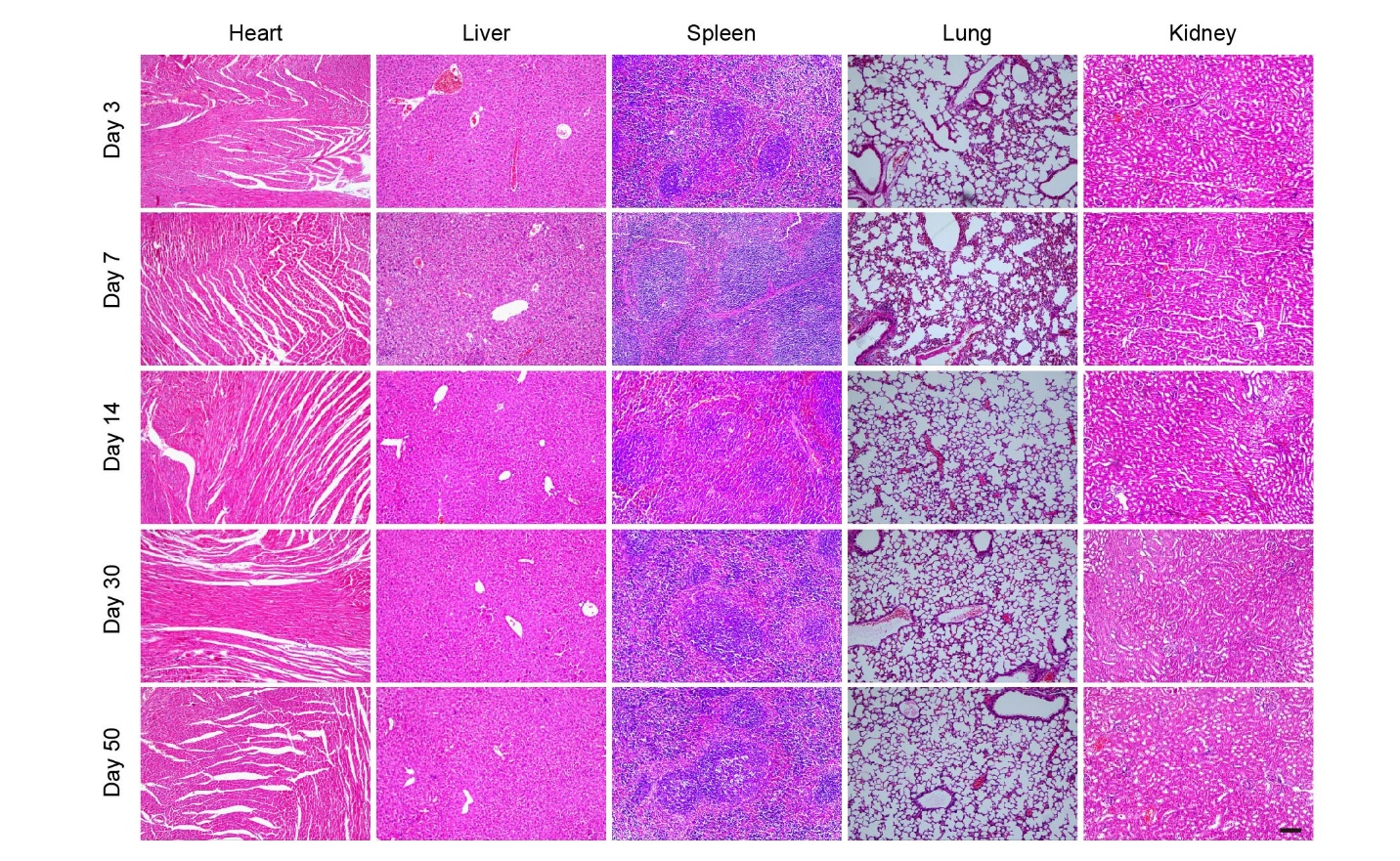
**

**FIGURE S8.** *In vivo* biosafety evaluation. There were no apparent histological changes in the heart, liver, spleen, lung, or kidney after hydrogels were injected for 3, 7, 14, 30, and 50 days. Scale bar: 100 μm.


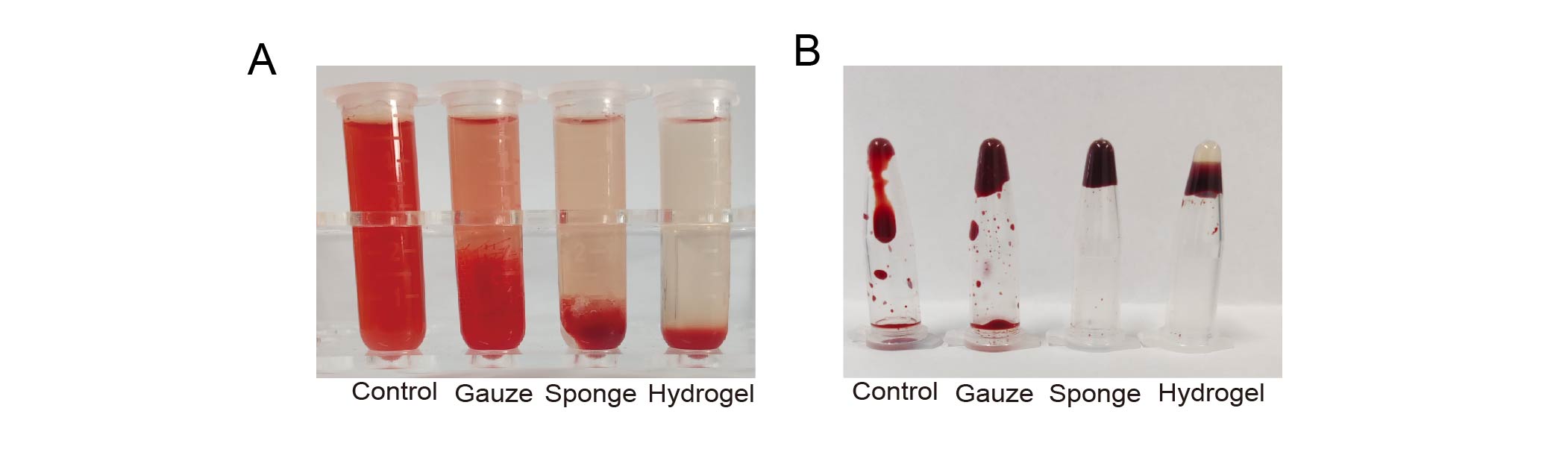


**FIGURE S9.** Whole blood clotting performance of hydrogels. (A) BCI. (B) Whole blood coagulation time.


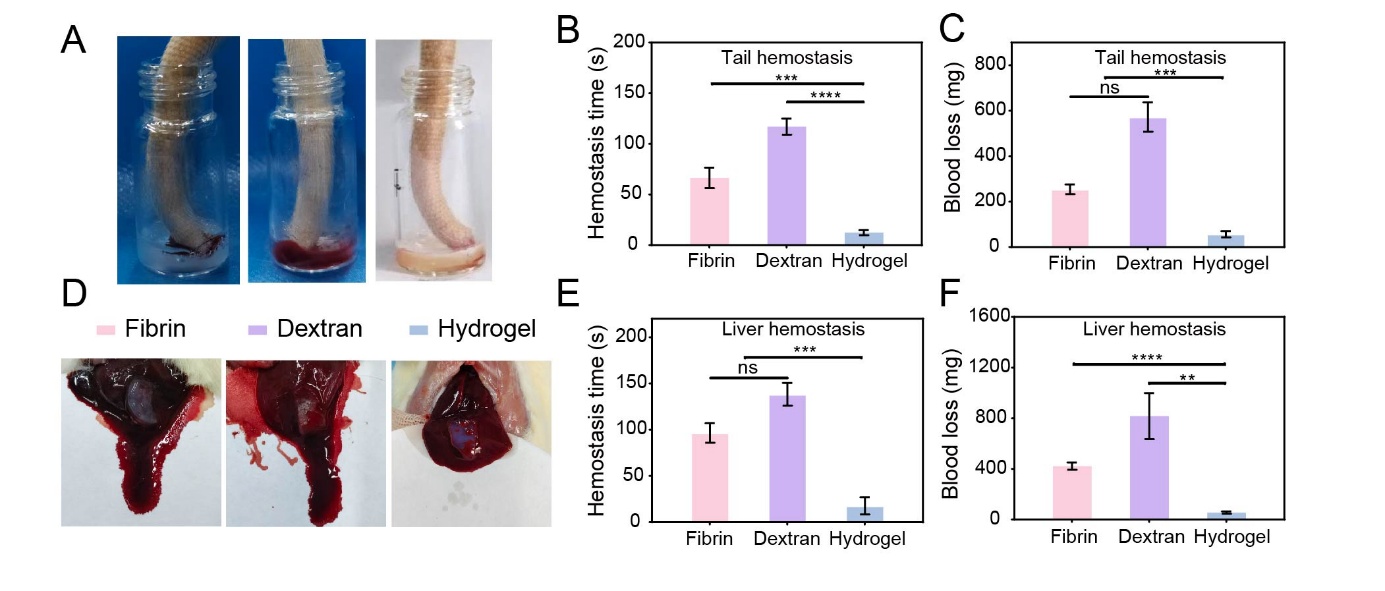


**FIGURE S10.** (A) Rat tail‑transection model. (B, C) Quantitative analysis of bleeding time and total blood loss for the Fibrin, Dex‑MA, and fibrin–dextran-MA–DMA (dual‑network hydrogel) groups. (D) Rat liver‑incision model. (E, F) Quantitative analysis of bleeding time and total blood loss for the same treatment groups. Data are presented as mean ± SD (n = 3). Statistical significance was assessed using Student’s t-tests and one-way ANOVA; ns, not significant, *P <0.05, **P <0.01, ***P <0.001 and ****P <0.0001.


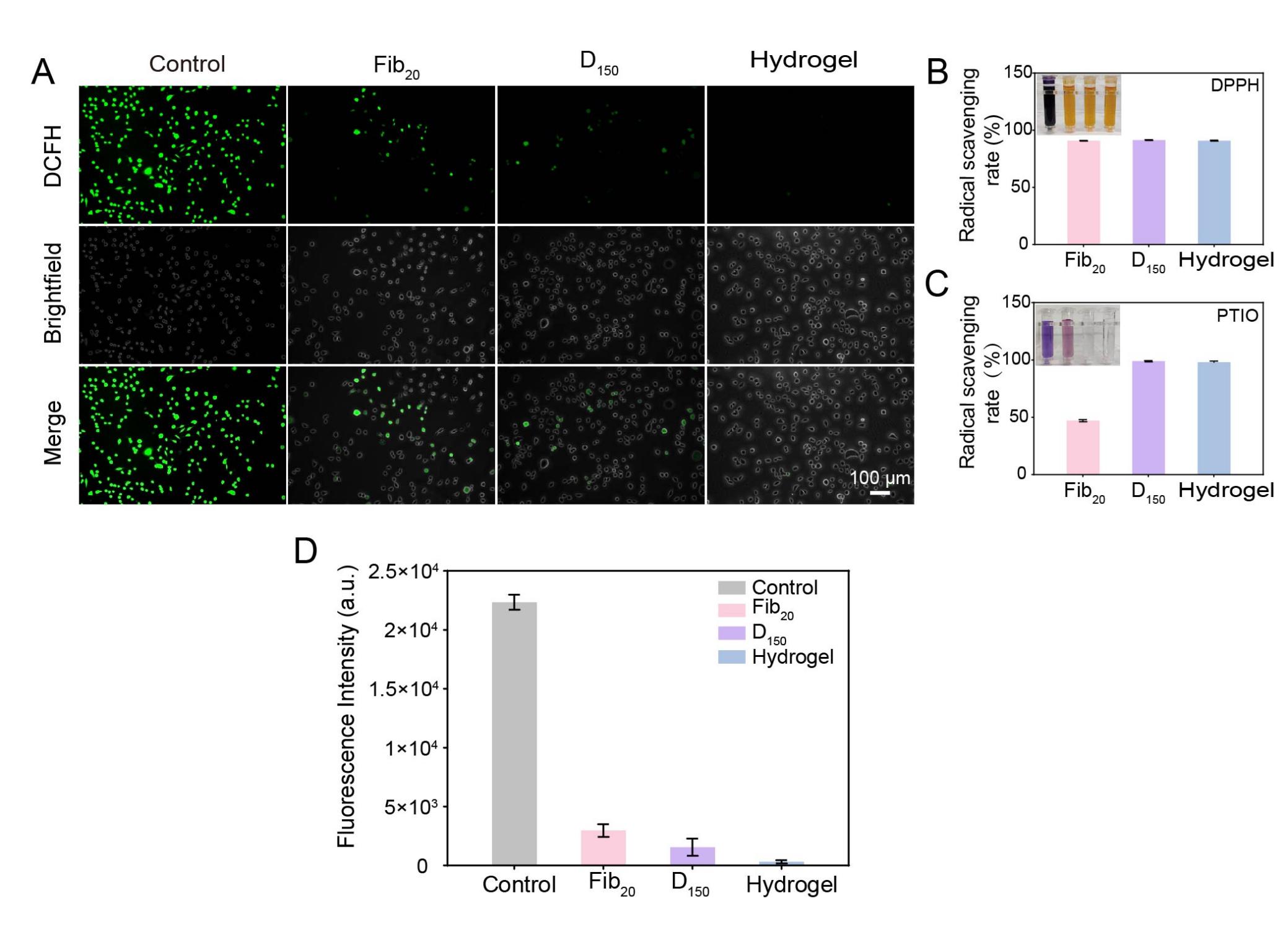


**FIGURE S11.** Antioxidant activities of hydrogels. (A) ROS in cells were detected by DCFH-DA staining. Scale bar: 100 μm. (B) DPPH radicals scavenging activity of hydrogels. (C) PTIO radicals scavenging activity in hydrogels. Fib_20_ (fibrinogen 20 mg/mL, DMA 1.86 mg/mL, thrombin 20 U/mL), D_150_ (dextran-MA 150 mg/mL, DTT 15 mg/mL, DMA 1.86 mg/mL). (D) Quantitative analysis of intracellular ROS levels. Fluorescence intensity is expressed in arbitrary units. Data are presented as mean ± SD (n = 3).


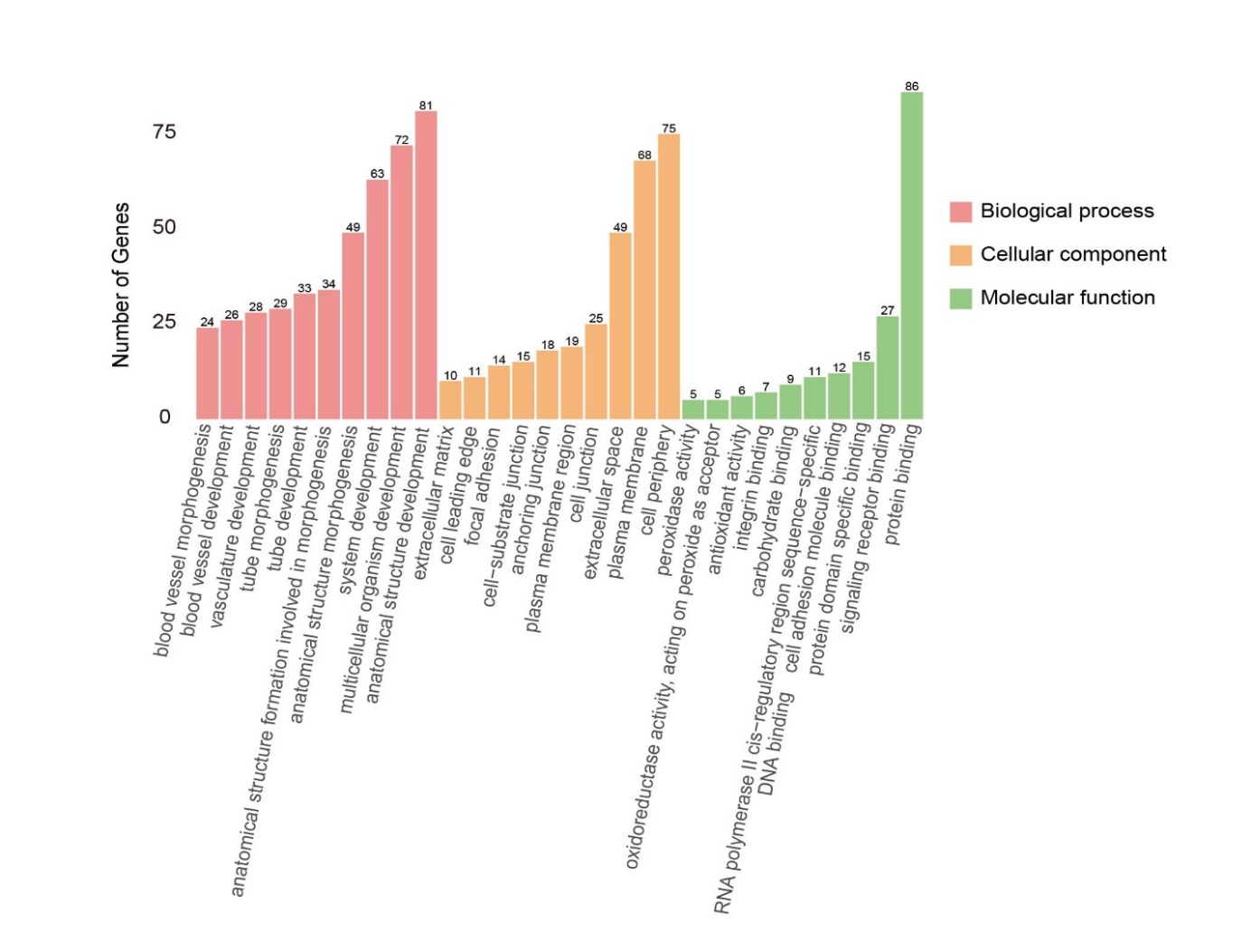


**FIGURE S12.** Enriched GO terms of DEGs in RAW264.7 cells with LPS and fibrin–dextran-MA–DMA hydrogel treatments, including BP, CC, and MF.


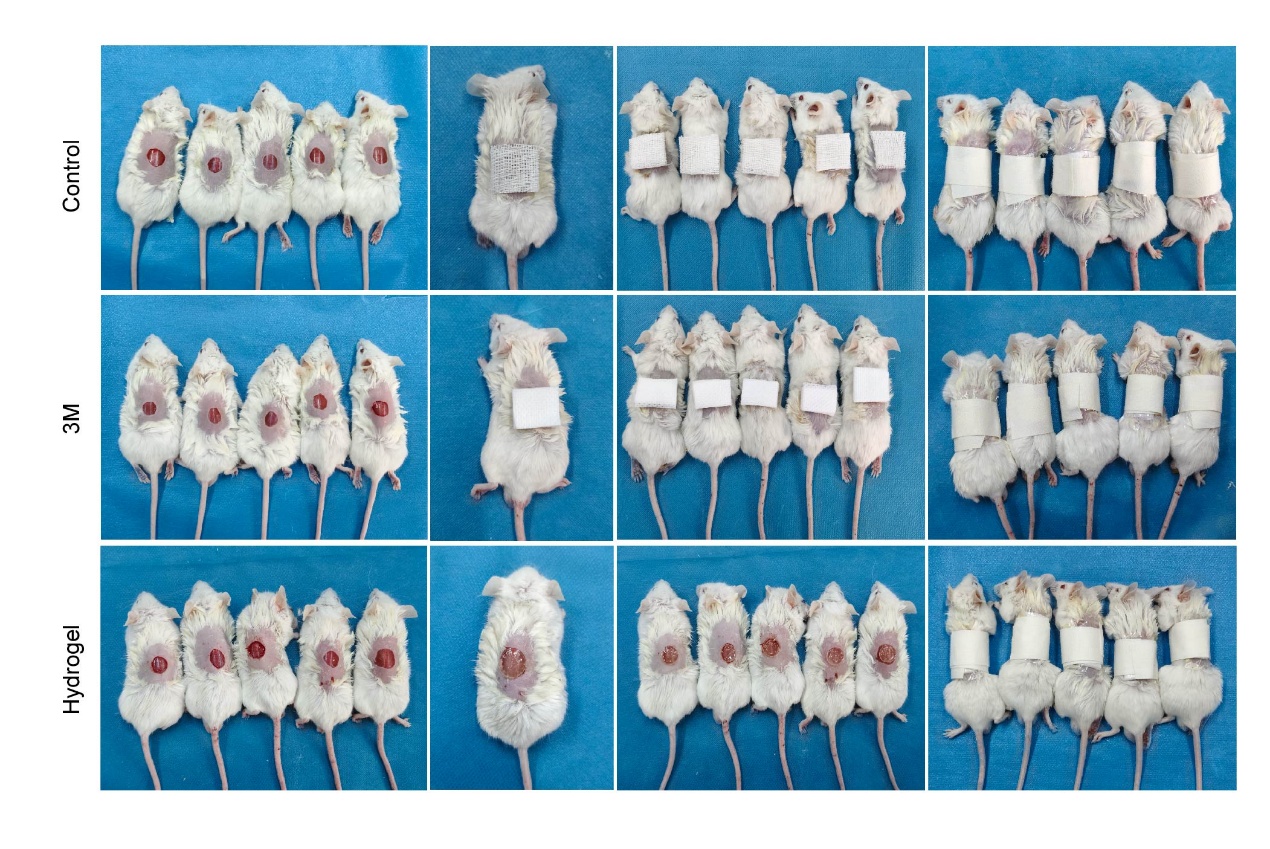


**FIGURE S13.** Wound creation. The mice were first bandaged with gauze, 3M, and hydrogel on day 0.


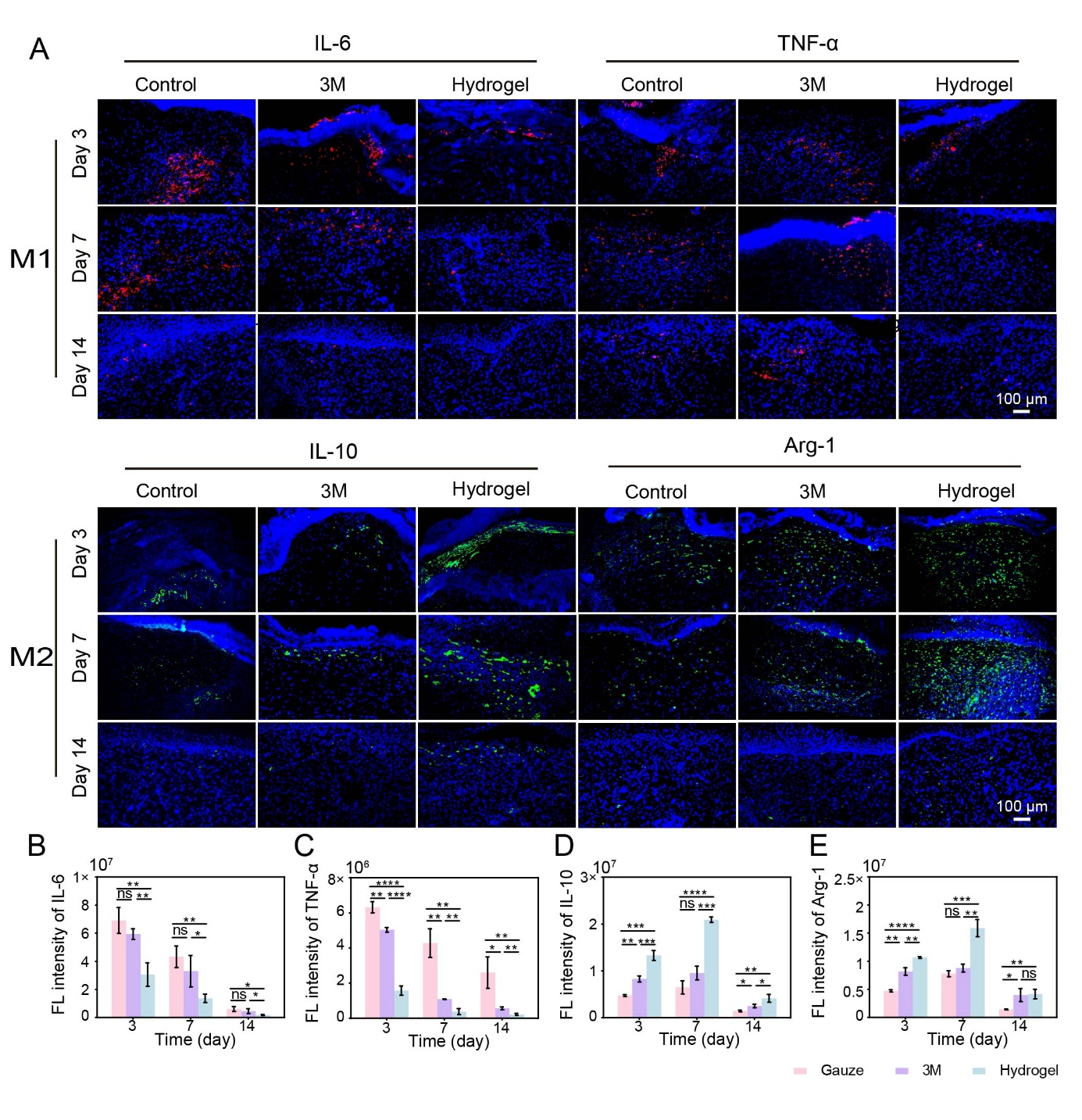


**FIGURE S14.** *In vivo* anti-inflammatory properties. (A) Immunofluorescence staining of IL-6, TNF-α, IL-10, and Arg-1 on days 3, 7, and 14. Scale bar: 100 μm. (B-E) Quantitative analysis of immunofluorescence intensity of IL-6, TNF-α, IL-10, and Arg-1 of different groups. Data are presented as mean ± SD (n = 3). Statistical significance was assessed using Student’s t-tests and one-way ANOVA; ns, not significant, *P <0.05, **P <0.01, ***P <0.001 and ****P <0.0001.


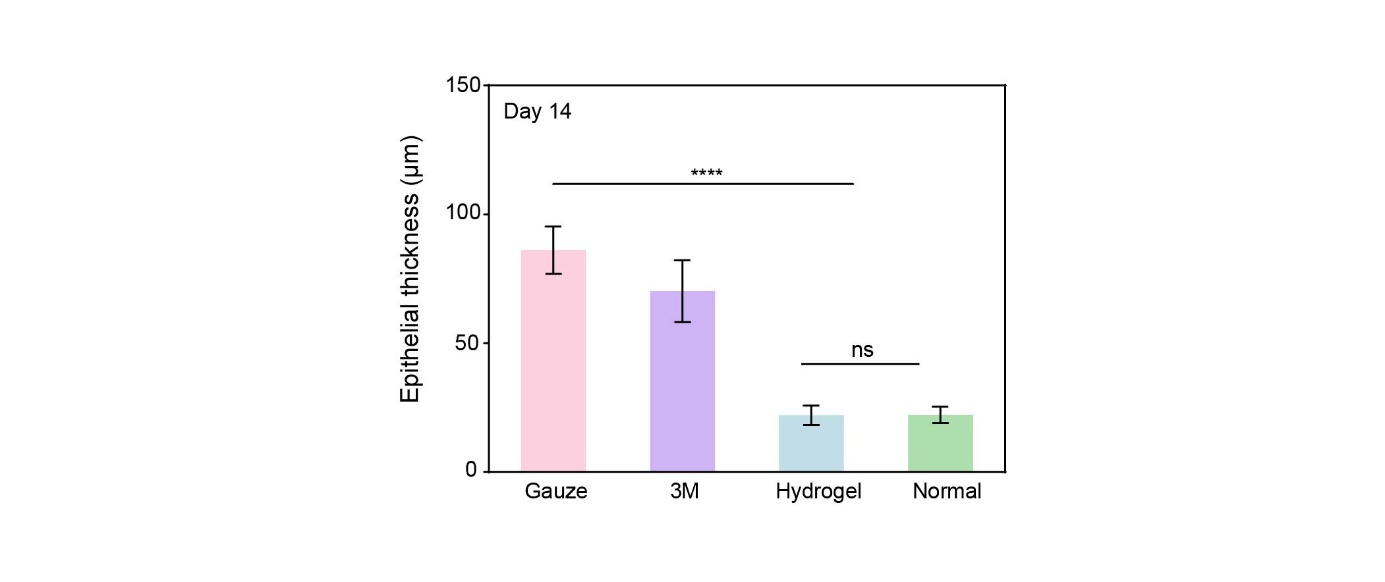


**FIGURE S15.** The thickness of the regenerated epithelium at the wound site from different dressing groups on day 14. Data are presented as mean ± SD (n = 3). Statistical significance was assessed using Student’s t-tests; ns, not significant, ****P <0.0001.

**TABLE S1.** Overview of fibrin−dextran-MA−DMA hydrogels.

| Hydrogel | fibrinogen [mg/mL] | dextran-MA [mg/mL] | thrombin [U/mL] | DMA [mg/mL] | DTT [mg/mL] |
| --- | --- | --- | --- | --- | --- |
| Fib_20_D_50_ | 20 | 50 | 20 | 1.86 | 5 |
| Fib_20_D_100_ | 20 | 100 | 20 | 1.86 | 10 |
| Fib_20_D_150_ | 20 | 150 | 20 | 1.86 | 15 |
| Fib_10_D_150_ | 10 | 150 | 10 | 1.86 | 15 |
| Fib_30_D_150_ | 30 | 150 | 30 | 1.86 | 15 |
| Fib_0_D_150_ | 0 | 150 | 0 | 1.86 | 15 |

**TABLE S2.** Primer sequences used for qRT-PCR.

| Target gene | Forward sequence (5’-3’) | Reverse sequence (5’-3’) |
| --- | --- | --- |
| IL-6 | CTTGGGACTGATGCTGGTG | GCCATTGCACAACTCTTTTCTC |
| TNF-α | CCACGCTCTTCTGTCTACTG | CTCCTCCACTTGGTGGTTTG |
| IL-10 | GCTGGACAACATACTGCTAACC | ATTTCCGATAAGGCTTGGCAA |
| Arg-1 | CAAGACAGGGCTCCTTTCAG | CTTATGGTTACCCTCCCGTTG |
| β-actin | GTTTGAGACCTTCAACACCCCAG | GGCGTGAGGGAGAGCATAG |
